# Non-invasive brain stimulation therapy on neurological symptoms in patients with multiple sclerosis: A network meta analysis

**DOI:** 10.3389/fneur.2022.1007702

**Published:** 2022-11-15

**Authors:** Xiaoyun Zhang, Yaping Huai, Zhiqiang Wei, Weiwei Yang, Qizhi Xie, Li Yi

**Affiliations:** ^1^Rehabilitation Department, Shenzhen Longhua District Central Hospital, Shenzhen, Guangdong, China; ^2^Shenzhen Longhua District Rehabilitation Medical Equipment Development and Transformation Joint Key Laboratory, Shenzhen, Guangdong, China; ^3^Neurology Department, Peking University Shenzhen Hospital, Shenzhen, Guangdong, China

**Keywords:** non-invasive brain stimulation, multiple sclerosis, neurological symptom, network meta-analysis, transcranial magnetic stimulation, transcranial direct current stimulation

## Abstract

**Objective:**

The aim of the study was to evaluate non-invasive brain stimulation (NIBS) [including transcranial magnetic stimulation (TMS) and transcranial electrical stimulation (tES)] on neurological symptoms in patients with multiple sclerosis (PwMS).

**Method:**

We searched PubMed, Embase, Cochrane Library, Web of Science and Ovid MEDLINE until February 2022. And we evaluated the included studies for methodological quality by the Cochrane bias risk assessment tool and assessed the studies' certainty of evidence using the Grading of Recommendations Assessment, Development and Evaluation (GRADE) framework. We performed network meta analysis (NMA) by using Stata 15 and ranked the results of the NMA by using the surface under the cumulative ranking curve (SUCRA) ranking chart.

**Result:**

Twenty seven clinical trials were finally included (*N* = 596, 66.4% women). For the immediate effects, rTMS over M1 yielded the most optimal scheme for fatigue reduction among all the interventions compared to the sham stimulation groups [MD = −0.85, 95% CI (−1.57, −0.14)] (SUCRA = 82.6%). iTBS over M1 yielded the most signifcant reduced pain level than the sham groups did [MD = −1.26, 95% CI (−2.40, −0.11)] (SUCRA = 98.4%). tDCS over F3 was the best protocol of NIBS to improve quality of life (QOL) [MD = 1.41, 95% CI = (0.45,2.36)] (SUCRA = 76.7%), and iTBS over M1 may significantly reduce spasticity compared to sham stimulation [MD = −1.20, 95% CI = (−1.99, −0.41)] (SUCRA = 90.3%). Furthermore, rTMS, tRNS, and tDCS on certain areas may improve PwMS accuracy, response time, manual dexterity, pain relief and QOL, but does not show statistically significant differences. The evidence assessed using GRADE is very low.

**Conclusion:**

Based on the NMA and SUCRA ranking, we can conclude that symptoms including fatigue, pain, spasticity, and QOL can be improved by following NIBS protocol after treatment. Nonetheless, most of the included studies lack a good methodology, and more high-quality randomized clinical trials are needed.

## Introduction

Multiple sclerosis (MS) is a chronic inflammatory and neurodegenerative disease with an unknown cause (1). It is also a permanent neurological disorder affecting young adults with the highest prevalence between the ages of 35 and 64 years regardless of race or ethnicity (2). Patients with multiple sclerosis (pwMS) have a 7 to 14 year reduction in life expectancy in comparison with the healthy population and approximately 50% died directly from its causes (3). A great deal of chronic neurological disability results from MS, including disorders of strength, sensation, coordination and balance, in addition to visual impairment, cognitive and affective deficits (4). Currently, there is no definitive cure for MS. Current treatment is based on immunosuppressive and immune-modulating medications as well as disease-modifying therapies (5). Even though significant advances have been made in the treatment of MS, disability progression and early mortality remain serious concerns (5). New approaches to neuropsychiatric symptoms are therefore needed for the MS population.

As new and developing techniques, non-invasive brain stimulation (NIBS) techniques provide relatively new therapeutic options which show promise in treating varieties of neurological disorders through reducing or modulating cortical excitability and plasticity (6). The two most common NIBS techniques are transcranial magnetic stimulation (TMS) and transcranial electrical stimulation (tES) (7). TMS induces a short electrical pulse on the cortex through the use of magnetic fields, specifically their rapid changes, which in turn generates action potentials over the cortical region of interest (8). There are numerous ways to use TMS, such as single pulses, repetitive TMS (rTMS) pulses, and intermittent theta burst stimulation (iTBS). On the other hand, tES works by passing a weak electric current between electrodes placed on the scalp, thereby stimulating the brain tissues between the electrodes. tES consists of transcranial direct current stimulation (tDCS), transcranial alternating current stimulation (tACS) and transcranial random noise stimulation (tRNS), depending on the type of electric current that is used (9). Among the tES techniques, tDCS is currently the most used, while the polarity of tDCS allows it to be further divided into anodal or cathodal (10).

The efficacy of TMS or tDCS (alone or in combination with conventional training) on the MS neuropsychiatric symptoms has been demonstrated in previous studies across a number of domains, including fatigue, motor performance, spasticity, pain, cognitive abilities, sensory deficit, bladder function and mood disorders (11–14). According to guidelines on rTMS, iTBS for the contralateral leg area of M1 (or both M1) has some potential for treating lower limb spasticity probable (Level B of evidence) (13). A meta-analysis of 14 studies and 207 patients found the 1.5 mA subgroup of tDCS and bilateral S1 subgroup of tDCS to be effective in fatigue reduction in PwMS, whereas TMS and tRNS did not outperform sham stimulation (12). Using data from 25 randomized controlled trials (RCTs) including 491 patients, Kan concluded that tDCS may improve cognitive function and reduce fatigue for people with multiple sclerosis, while rTMS may relieve muscle spasticity (14). Neither adverse side effects nor deleterious pharmacotherapy interactions were reported (12, 14, 15). In summary, TMS and tDCS may be safe and appealing as part of the armamentarium in addition to conventional therapy.

However, although reviews and meta-analyses have attempted to summarize the latest evidence on the therapeutic effects of NIBS on MS symptoms, few studies have compared different NIBS techniques and protocols to reach the most efficient treatment for MS neurological symptoms. In addition, due to the limited number of clinical trials for emerging NIBS techniques, including transcranial alternating current stimulation (tACS) and transcranial focused ultrasound (tFUS), previous reviews may not fully reflect the effects of NIBS on MS. And thus, we conducted this network meta analysis (NMA) to include the latest research and evaluate the effect of different NIBS on the common symptoms and disabilities in pwMS.

## Materials and methods

The meta analysis was registered with PROSPERO No. CRD42022342409 and complied with the PRISMA statement (16).

### Search strategy and exclusion criteria

The authors searched five electronic databases (Pubmed, EMBASE, Cochrane Central Register of Controlled Trials, Web of Science, and Ovid) up to February 2022. The detailed search strategy is shown in Supplementary Table 1.

Based on the PICOS tool, all enrolled studies followed the criteria: (1) Population: people with multiple sclerosis; (2) Intervention: non-invasive brain stimulation; (3) Comparator: sham stimulation; (4) Outcomes: neurological symptoms and disabilities including cognition, fatigue, spasticity, pain, manual performance, walking performance, and quality of life (QOL); (5) Study type: clinical trials. Studies were excluded if they were (1) Studies with incomplete, unreported or incorrected data; (2) Studies from reviews, letters, case reports, animal studies, protocols, conference abstracts or correspondence; (3) Duplicated studies.

### Study selection and data extraction

The literature was screened, selected and processed using the literature management software Endnote X9. Two investigators (XYZ and ZQW) independently evaluated the studies for inclusion, extracted data from the articles, and assessed their bias risk. A standardized and pre-defined data extraction form (Supplementary Table 2) was used to collect data under the following headings: (1) author, (2) year of publication, (3) country, (4) sample size, (5) number of male or female, (6) mean age, (7) intervention, (8) control group, (9) outcome indicators and (10) details of the interventions. Any disagreement was adjudicated by discussion or consulting the corresponding author.

### Data analysis

We performed the NMA on STATA version 15.0 (StataCorp LLC, College Station, TX, USA). All variables included in our review were continuous variables and for continuous data, we estimated the summary standardized mean difference (SMD) with 95% confidence intervals (CI) in situation of different kinds of rating scales or tests and the mean difference (MD) with CI in situation of uniform scales or tests in individual outcomes (17).

As for the analytical procedure of this study, we employed a mixed comparison with generalized linear mixed model to analyze the direct and indirect comparisons among the NMA (18). In the generated network diagrams, each node represented a different NIBS technique or the sham stimulation, the lines connecting the nodes showed head-to-head comparisons between interventions. The node's size and lines' width were proportional to the number of studies.

Surface under the cumulative ranking curve (SUCRA), one of the quantitative ranking methods was used. In expressing the effectiveness or acceptability of interventions, SUCRA is a useful indicator.

### Risk of bias and level of confidence

Two authors (XZ and WY) independently assessed the risk of bias (ROB), in accordance with the Cochrane Collaboration software, Review Manager (RevMan) 5.3. The following seven domains were considered: (1) randomized sequence generation, (2) treatment allocation concealment, blinding of (3) participants and (4) personnel, (5) incomplete outcome data, (6) selective reporting and (7) other sources of bias. The overall risk of bias was categorized into three groups: low, high, and unclear risk of bias (19).

In order to check for publication bias caused by small-scale studies, we drawn a network funnel plot of each group of studies and examined it visually using the symmetry criterion.

With Grading of Recommendations Assessment, Development, and Evaluation (GRADE) framework, we additionally evaluated the certainty of evidence contributing to network estimates for the main outcomes (20).

## Results

### Study selection

**Figure 1:**
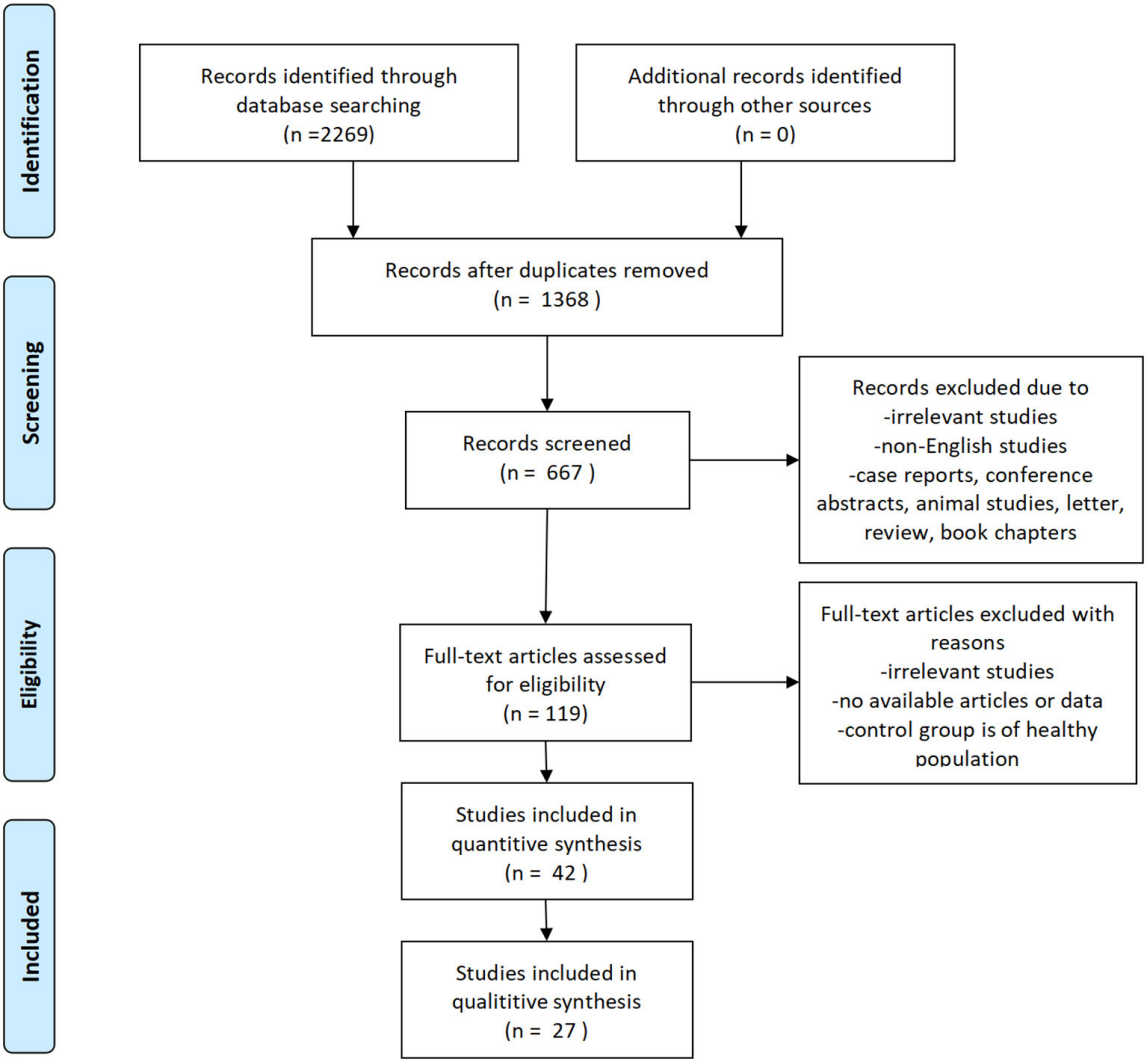
Flow diagram of literature selection.

We searched the databases of PubMed, Embase, Web of Science, Ovid, Cochrane Library up to Febray 18th, 2022. No language restriction was imposed. In addition, we manually searched for potentially eligible articles cited in relevant review articles and meta-analyses. There were a total of 2,269 documents retrieved from the electronic database, and no additional documents were identified through other resources. After duplicated documents were deleted by EndNote X9 software (Thomson ResearchSoft, Stanford, CT, USA), 1,605 documents were excluded because they failed to meet the inclusion criteria and the 667 titles and abstracts of the remaining documents were read. The remaining 119 documents were read in full and 77 documents were excluded (for the reasons including: irrelevant studies, no available articles or data, control group is of healthy population), leaving 42 articles to be included. The results and data were extracted from the full text of the 42 articles and reclassified into different subgroup of symptoms. Only data with more than two interventions were eligible for network meta analysis. Eventually 27 articles (21–43) were included into the final analysis (Figure 1).

### Quality assessment and GRADE

**Figure 2:**
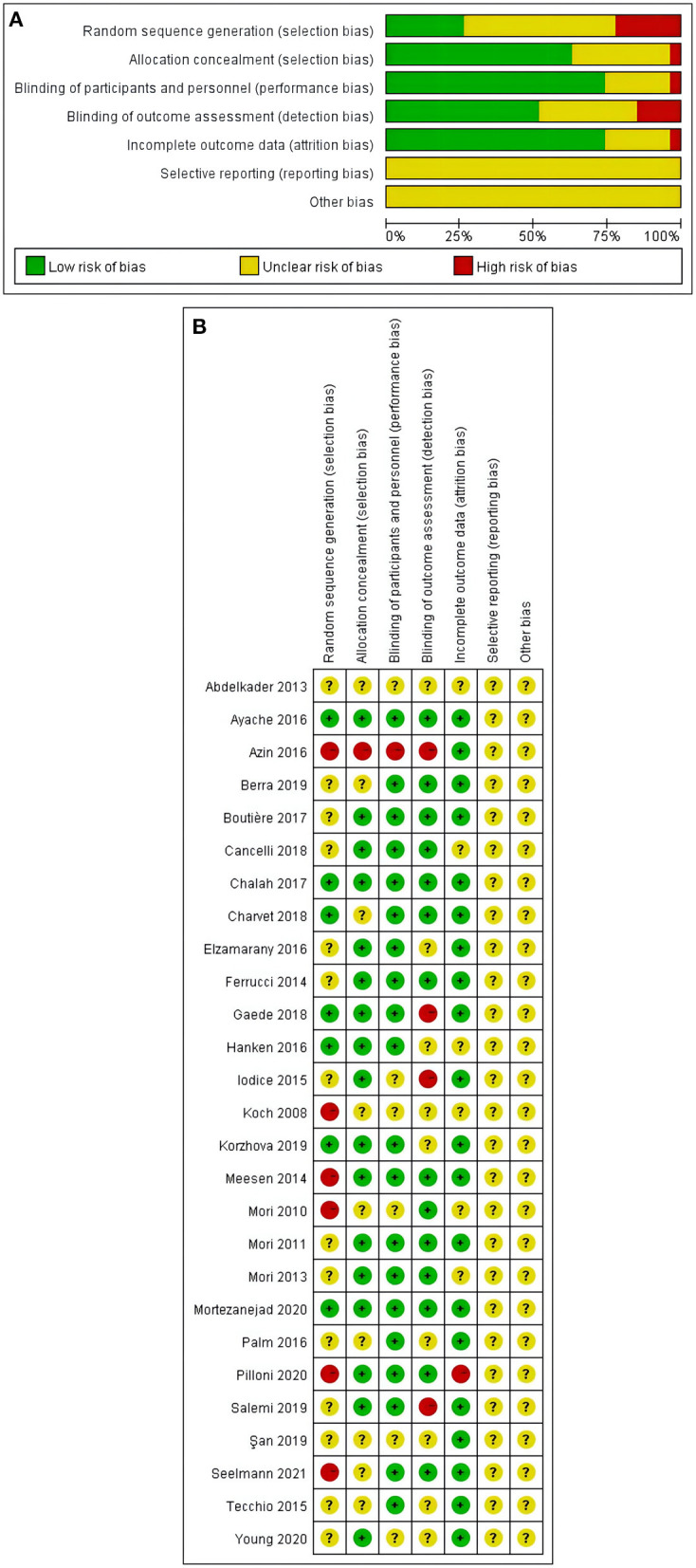
**(A,B)** Risk of bias assessment summary according to the Cochrane risk of bias tool: Red, green, and yellow colors indicates high, low, and unclear risk of bias, respectively. **(A)** Risk of bias summary: review authors' judgements about each risk of bias item for each included study. **(B)** Risk of bias graph: review authors' judgements about each risk of bias item presented as percentages across all included studies.

Results for the 7 items of the Cochrane Risk of Bias tool for the 27 studies were reported in Figures 2A,B. Nine studies had more than one item with high risk of bias (21, 22, 26, 27, 36, 38, 41, 43, 44), and 18 studies had one or more items rated as unclear risk of bias (23–25, 28–37, 39, 40, 42, 45–47).

Selection bias and detection bias were found to be the top two risk factors. Six study was assessed to have a high risk of selection bias due to pseudo-randomized in the study (21, 22, 26, 41, 43, 44). Three studies were rated as high risk of detection bias reporting as single-blind study (27, 36, 38). More details can be found in Figures 2A,B.

We incorporated the GRADE judgments in Supplementary Tables 3, 4. For all comparisons, the certainty of evidence for treatment effects of efficacy was very low because of study limitation, indirectness and inconsistency.

### Characteristics of the included studies

In total, we included data from 27 clinical trials, which included 596 patients (Female 396, 66.4%) diagnosed with MS. The NIBS techniques explored were tDCS (*N* = 14) (24–28, 30, 33–35, 40–43, 46), rTMS (*N* = 5) (21, 29, 36, 39, 45), iTBS (*N* = 5) (22, 23, 32, 37, 44), tRNS (*N* = 2) (31, 38) and transcutaneous spinal direct current stimulation (ts-DCS) (*N* = 1) (47). The targets were the primary motor cortex (M1) (*N* = 16) (21–23, 25–27, 29, 34, 37–39, 41–43, 45–47), the left dorsolateral pre-frontal cortex (DLPFC or F3) (*N* = 5) (31, 33, 35, 40, 46) and primary somatosensory cortex (S1) (*N* = 3) (24, 28, 34). Countries or regions of the original studies included Italy (*N* = 10) (21–25, 27, 28, 34, 38, 47), France (*N* = 4) (31–33, 46), Germany (*N* = 3) (30, 36, 43), Egypt (*N* = 2) (29, 45), Iran (*N* = 2) (40, 44), and USA (*N* = 2) (35, 41). Detailed information can be found in Table 1.

**Table 1:** Characteristics of the studies included in the meta-analysis.

**References**	**Year of** **publication**	**Country** **or region**	**Sample** **size**	**Male/** **Female**	**Age (mean+SD)**	**Intervention**	**Control**	**Outcome** **indicators**	**Experiment group**		
									**Electrode** **placement**	**Intensity**	**Area**
Abdelkader et al. (45)	2013	Egypt	21	12/9	UA	rTMS	rTMS of low frequency	Spasticity	M1	5Hz	/
Ayache et al. (46)	2016	France	16	3/13	A+C: 48.9 ±10.0	tDCS	Sham control	Pain, attention, mood, fatigue	F3	2 mA	25 cm^2^
Azin et al. (44)	2016	Iran	36	9/27	A: 30.8 ± 6.1; C: 29.7 ± 7.9	iTBS	Sham control	Manual dexterity, reaction time, response accuracy	M1	5 Hz	/
Berra et al. (47)	2019	Italy	33	8/25	A: 57.6 ± 9.1; C: 54.0 ± 7.79	ts-DCS	Sham control	Pain, fatigue, spasticity	TSC	2 mA	35 cm^2^
Boutière et al. (32)	2017	France	17	9/8	A: 48.2 ± 9.4; C: 55.4 ± 11.1	iTBS	Sham control	Spasticity	M1	10 bursts, three stimuli at 50 Hz, repeated at a theta frequency of 5 Hz every 10 s, for a total of 600 stimuli (192 s).	/
Cancelli et al. (34)	2018	Italy	10	2/8	A+C: 43.2 ± 13.1	tDCS	Sham control	Fatigue	S1	1.5mA	35 cm^2^
Chalah et al. (33)	2017	France	10	6/4	A+C: 40.50 ± 11.18	tDCS	Sham control	Fatigue, attention	F3	2 mA	25 cm^2^
Charvet et al. (35)	2018	USA	35	12/23	A: 44.8 ± 16.2; C: 43.4 ± 16.2	tDCS	Sham control	Fatigue, depression	F3	2 mA	25 cm^2^
Elzamarany et al. (29)	2016	Egypt	24	15/9	A+C: 22.97 ± 9.04	rTMS	Sham control	Manual dexterity	M1	5 Hz	/
Ferrucci et al. (25)	2014	Italy	15	4/11	A+C: 40.3 ± 2.3	tDCS	Sham control	Fatigue	M1	1.5mA	35 cm^2^
Gaede et al. (36)	2018	Germany	19	5/14	A: 47 ± 14.07; C: 41± 4.44	rTMS	Sham control	Fatigue, depression	PFC	5 Hz	/
Iodice et al. (27)	2015	Italy	20	5/15	A: 43.3 ± 7.5; C: 40.3 ± 4.5	tDCS	Sham control	Spasticity	M1	2 mA	35 cm^2^
Koch et al. (21)	2008	Italy	8	3/5	UA	rTMS	Sham control	Manual dexterity	M1	5 Hz	/
Korzhova et al. (37)	2019	Russia	34	14/20	UA	iTBS	Sham control	Fatgue, spasticity, pain	M1	35Hz	/
Meesen et al. (26)	2014	Belgium	31	9/22	A+C: 48.16 ± 10.13	tDCS	Sham control	Attention, fatigue, pain, sleep quality	M1	1 mA	25 cm^2^
Mori et al. (22)	2010	Rome	20	7/13	A+C: 44.3 ± 12.5	iTBS	Sham control	Spasticity	M1	10 bursts, three stimuli at 50 Hz, repeated at a theta frequency of 5 Hz every 10 s, for a total of 600 stimuli (200 s).	/
Mori et al. (23)	2011	Rome	20	13/7	A+C: 38.4 ± 11.3	iTBS	Sham control	Fatigue, spasticity, QOL	M1	10 bursts, three stimuli at 50 Hz, repeated at a theta frequency of 5 Hz every 10 s, for a total of 600 stimuli (200 s).	/
Mortezanejad et al. (40)	2020	Iran	36	6/30	A+C: 32.617 ± 6.345	tDCS	Sham control	Fatigue, QOL	F3	1.5 mA	35 cm^2^
Palm et al. (31)	2016	France	16	3/13	A+C: 47.4 ± 8.9	tRNS	Sham control	Pain, attention, mood	F3	2 mA	25 cm^2^
Pilloni et al. (41)	2020	New York	15	4/11	A: 52.1 ± 12.9; C: 53.7 ± 9.8	tDCS	Sham control	Walking functions	M1	2.5 mA	25 cm^2^
Salemi et al. (38)	2019	Italy	17	5/12	A: 39.8 ± 10.4; C: 44.2 ± 7.3	tRNS	Sham control	Fatigue, QOL	M1	1.5 mA	25 cm^2^
San et al. (39)	2019	Turkey	16	8/8	A+C: 49.93 ± 12.27	rTMS	Sham control	Fatigue, spasticity, cognition, QOL	M1	5 Hz	/
Seelmann-Eggebert et al. (43)	2021	Germany	16	10/6	A+C: 51.2 ± 10.6	tDCS	Sham control	Accuracy	M1	1mA	25 cm^2^
Tecchio et al. (28)	2015	Italy	13	4/9	A+C: 45.8 ± 7.6	tDCS	Sham control	Fatigue, manual dexterity	S1	1.5 mA	35 cm^2^
Young et al. (42)	2020	Melbourne	30	6/24	A: 51.2 ± 9.3; C: 49.87 ± 12.9	tDCS	Sham control	Pain, QOL	M1	2 mA	35 cm^2^
Hanken et al. (30)	2016	Germany	48	11/37	A+C: 49.08 ± 9.46	tDCS	Sham control	Fatigue	P4	1.5 mA	35 cm^2^
Mori et al. (24)	2013	Italy	20	7/13	A+C: 44.3 ± 12.5	tDCS	Sham control	QOL	S1	2 mA	35 cm^2^

A, active group; C, control group with sham stimulation; UA, unavailable; rTMS, repetitive transcranial magnetic stimulation; tDCS, transcranial direct current stimulation; tRNS, transcranial random noise stimulation; iTBS, intermittent theta burst stimulation; ts-DCS, transcutaneous spinal DCS; QOL, quality of life; M1, primary motor cortex; TSC, thoracic spinal cord; S1, primary somatosensory cortex; P4, right parietal cortex; F3, left dorsolateral prefrontal cortex; PFC, prefrontal cortex.

Outcomes indicators included the following seven domains: accuracy, reaction time, fatigue, pain, spasticity, manual dexterity and QOL. In studies using several scales to evaluate a certain symptom, we selected the most frequently used one. For trials with immediate and follow-up outcomes, effects immediately after the last session of NIBS and those after the last session of follow-up were examined separately. Eventually, we assessed the seven domains in the analysis of immediate effect of NIBS (accuracy, reaction time, fatigue, manual dexterity, pain, spasticity and QOL) and two domains (fatigue and spasticity) in the analysis of follow-up effect, see Supplementary Table 5.

### Network meta-analysis

Network plots showed the different treatments that were compared in the network meta-analysis. As the intermediate effect illustrated in Supplementary Figure S1 and the long durable effect shown in Supplementary Figure S2.

#### Immediate effect

##### Accuracy

The results of the NMA showed that relative to the sham stimulation, tRNS over F3 [MD = 0.58, 95% CI = (−0.38,1.54)], tDCS over right PPC [MD = 0.59, 95% CI = (−0.52,1.70)], tDCS over M1 [MD = 0.18, 95% CI = (−0.64,1.01)] were superior to the sham group in improving accuracy. However, the statistical significance was not significantly among the difference between tDCS or tRNS treatment versus sham stimulation. The details were shown in Supplementary Table 3A. And according to the SUCRA results, tRNS over F3 yielded the most increased in accuracy in MS patients among all the interventions (SUCRA = 77.6%).

##### Reaction time

A comparison of the NMA results revealed that tRNS over M1 [MD = −0.61, 95% CI = (−3.27, 2.05)] and iTBS over M1 [MD = −0.54, 95% CI = (−3.12, 2.03)] reduced reaction times better than sham stimulation. Although there were no statistically significant differences observed for the outcomes. Further details were shown in Supplementary Table 3B. And according to the SUCRA results, tRNS over M1 yielded the most decrease in reaction time in MS patients among all the interventions (SUCRA = 61.7%).

##### Fatigue

The NMA showed that rTMS over M1 [MD = −0.85, 95% CI (−1.57, −0.14)] and tDCS over F3 [MD = −0.67, 95% CI = (−1.18,−0.16)] had a better effect on reducing fatigue than sham stimulation and the above differences were statistically significant. Other protocols including tRNS over M1 [MD = −0.72, 95% CI = (−1.87, 0.42)], tDCS over S1 [MD = −0.58, 95% CI = (−1.30, 0.14)], tsDCS over thoracic spinal cord (TSC) [MD = −0.46, 95% CI = (−1.36, 0.44)], tDCS over P4 [MD = −0.41, 95% CI = (−1.15, 0.34)], tDCS over M1 [MD = −0.33, 95% CI = (−0.76, 0.10)], iTBS over M1 [MD = −0.26, 95% CI = (−0.96, 0.44)], tRNS over F3 [MD = 0.07, 95% CI = (−0.82, 0.97)] were superior in reducing fatigue compared to the sham group as well, but the results were not significant. The details are presented in Supplementary Table 3C. According to the SUCRA results, the most significant reduction in fatigue was observed among all interventions when rTMS was applied over M1 (SUCRA = 82.6%).

##### Manual dexterity

The main result revealed that rTMS over M1 [MD = −0.50, 95% CI = (−1.35, 0.35)] and iTBS over M1 [MD = −0.14, 95% CI = (−1.15, 0.86)] were superior to the sham group in improving manual dexterity despite no significant differences statistically. More details were shown in Supplementary Table 3D. And according to the SUCRA results, rTMS over M1 was associated with better improvement in manual dexterity when compared with other interventions (SUCRA = 81.6%).

##### Pain

Based on the NMA results, it appears that iTBS over M1 (MD = −1.26, 95% CI = (−2.40, −0.11)] may significantly reduce pain compared to a sham control group. Other protocols such as tDCS over M1 [MD = −0.18, 95% CI = (−1.27, 0.91)], tDCS over F3 [MD = −0.16, 95% CI = (−1.37, 1.04)] and tsDCS over TSC [MD = −0.06, 95% CI = (−1.04, 0.92)] were associated with lower pain levels than the sham stimulation groups, but that difference did not reach statistical significance, as suggested in Supplementary Table 3E. The results of the SUCRA study show that iTBS over M1 caused the most significant reduction in pain level among all interventions (SUCRA = 98.4%).

##### QOL

Among the main findings of the NMA, tDCS over F3 [MD = 1.41, 95% CI = (0.45, 2.36)] was superior to the sham group in terms of improving QOL, without any statistically significant differences. Other protocols including tRNS over M1 [MD = 0.44, 95% CI = (−0.52, 1.41)], tDCS over S1 [MD = 0.25, 95% CI = (−0.63, 1.13)] and tDCS over M1 [MD = 0.51, 95% CI = (−0.12, 1.15)] improved QOL more than the sham group despite no significant differences statistically. And the details were shown in Supplementary Table 3F. Among all interventions, tDCS over F3 caused the most significant improvement in QOL in the SUCRA study (SUCRA = 76.7%).

##### Spasticity

The results of the NMA showed that relative to the sham stimulation, iTBS over M1 [MD = −1.20, 95% CI = (−1.99, −0.41)], reduce spasticity with a statistical difference. While other protocols of NIBS including rTMS over M1 (HF) [MD = −0.83, 95% CI = (−1.88, 0.21)], tDCS over M1 [MD = −0.20, 95% CI = (−1.72, 1.31)] and tsDCS over TSC [MD = 0.04, 95% CI = (−1.38, 1.45)] were superior to the sham group in reducing spasticity despite no statistical differences. More details were shown in Supplementary Table 3G. The results of the SUCRA study show that iTBS over M1 caused the most significant decreasing spasticity among all interventions (SUCRA = 90.3%).

#### Longer durable effects (≥1 months)

##### Fatigue

The results of the NMA showed that tDCS over F3 [MD = −1.19, 95% CI = (−3.11, 0.74)], rTMS over M1 [MD = −0.84, 95% CI = (−2.33, 0.65)], tsDCS over TSC [MD = −0.87, 95% CI = (−2.92, 1.17)], tRNS over M1 [MD = −0.46, 95% CI = (−2.60, 1.69)], iTBS over M1 [MD = −0.38, 95% CI = (−2.33, 1.57)] and tDCS over M1 [MD = −0.08, 95% CI = (−1.32, 1.16)] were superior to the sham group in terms of relieving fatigue without statistically significant differences, and the details were shown in Supplementary Table 4A. The results of the SUCRA study show that tDCS over F3 caused the most significant decreasing in fatigue among all (SUCRA = 73.0%).

##### Spasticity

The results of the NMA showed that tsDCS over TSC [MD = −10.75, 95% CI = (−13.63, −7.86)] and iTBS over M1 [MD = −1.21, 95% CI = (−1.85, −0.57)] were superior to the sham group in decreasing spasticity significantly. Whereas tDCS over M1 [MD = −0.47, 95% CI = (−1.44, 0.50)] and rTMS over M1 [MD = −0.41, 95% CI = (−1.03, 0.21)] decreased spasticity with no statisitically significance when compared to the sham group, and the details were shown in Supplementary Table 4B. SUCRA showed that tsDCS over TSC led to the greatest reduction in spasticity among all (SUCRA = 100%).

### Publication bias test

Separate funnel plots were constructed for all outcome indicators to test for publication bias. A visual examination of the funnel plots did not reveal any significant publication bias for all the outcome indicators. Details were shown as in Supplementary Figures S3, S4.

## Discussion

Our study is the first NMA to compare different NIBS interventions for improving neuropsychiatric disabilities in pwMS. We analyzed 27 studies involving 596 people with MS. Results showed that in the short term, both rTMS over M1 and tDCS over F3 were more effective in reducing fatigue when compared to the sham group and iTBS over M1 was the most superior protocol in terms of spasticity reduction. While in the long-term run, tDCS over TSC and iTBS over M1 were the two best approaches for the reduction of spasticity. In addition, the use of TMS, RMS, and tDCS on certain target areas may be beneficial for patients' accuracy, reaction time, manual dexterity, pain and QOL with no discernible statistical differences.

As mentioned in the Introduction section, previous reviews have demonstrated the efficacy of anodal tDCS over left DLPFC and bilateral S1 for fatigue, as well as HF-rTMS and iTBS over M1 for muscle spasticity (12–14). Our findings are generally in line with those described previously. In addition, our work has yielded other significant findings.

First, our study showed the significant efficacy of rTMS over M1 and tDCS over F3 in the immediate treatment of fatigue, and we found rTMS over M1 reduced fatigue most significantly among all interventions. The most common complaint of patients with multiple sclerosis (PwMS) is fatigue (48). Previous study noted MS fatigue may relate to functional imbalance between homologous sensorimotor regions of the two hemispheres, and thus the effect of rTMS over M1 for the treatment of fatigue may be credited to the changes of neuromodulation to the inter-hemispheric interaction of primary sensorimotor areas of the brain (49). Pathogenesis of fatigue in PwMS may also include changes in cortico-striato-thalamo-cortical loops, increased energy demand, structural and functional neurological alterations and Adibi et al. (50). The mechanism behind the role of F3 in fatigue relief remains to be elucidated. Perhaps the reason lies in the hypothesis that F3 functions as a one of the hubs between the cortico-striato-thalamo-cortical loops (51).

Second, our study suggested that iTBS over M1 was found to be effective for both short-term and long-term spasticity treatment in our studies. The therapeutic effect of electromagnetic stimulation (rTMS or iTBS) on the affected brain area have been previously summerized by Naro et al. (52). Naro also reviewed that spasticity is usually caused by damage to nerve pathways within the brain or spinal cord (52). By enhancing corticospinal tract excitability and reducing stretch reflex, rTMS on the motor cortex may enhance spasticity management through the modulation of spinal excitability (53). Moreover, our study also found that tsDCS over TSC is significant in the long-term management of spasticity for PwMS. There is no clear mechanism for it and we can hardly found evidence of tsDCS on the relieve of spasticity in MS patients in other reviews. Though we found evidence of early and persistent clinical efficacy of tsDCS on central neuropathic pain for PwMS, probably through modulation of spinal nociception (47). Further studies are needed to determine how neuromodulation affects spinal cord function in PwMS.

Third, iTBS over M1 was proved to be the best way to alleviate pain for pwMS in our study. Pain is a frequent symptom experienced by up to 75 percent of MS patients (54). Although the mechanisms of MS pain are not fully understood, descending pain modulatory system dysfunction has been linked to human chronic pain conditions (54). And in patients with MS, the processes of brain networks that mediate pain relief may be disrupted in part (55). Stimulation on M1 is supposed to induce analgesic effects through an antidromic top-down modulation of thalamo-cortical pathways (55). In addition, previous study found that in healthy volunteers theta-burst stimulation (TBS) seemed more analgesic than high frequency repetitive transcranial magnetic stimulation and a higher number of pulses of prolonged continuous TBS might result in stronger analgesic effects (56).

In addition, our study found that tDCS over F3 was the best protocol among all the NIBS to improve QOL. MSQOL-54 is frequently used in pwMS to assess QOL (57). There are 12 subscales of MSQOL, which mainly involve physical functions and emotional wellbeings. Anodal tDCS acting on the patients' F3 region may alleviate left-hemispheric hypoactivation in the prefrontal cortex and improve psychological function and improve patients' QOL (58).

Although no definite conclusion was reached on the effects of NIBS on other symptoms in PwMS including accuracy, reaction time and manual dexterity, etc. in our study. Our study suggested that interventions targeting on F3 might help improve accuracy and QOL for MS patients in the short term, and might help relieve fatigue in the long run. And techniques targeting M1 could benefit not only fatigue, spasticity and pain as previously described, but might also improve reaction time and manual dexterity. The underlying etiology of fatigue, cognitive impairment, and pain in MS may be similar, and the overlap may be a result of lesions in MS occurring in highly interconnected deep gray matter, whereby their ripple effects may affect a variety of other brain circuits (59). It has been proposed that PwMS with impairment of hand performance may be correlated with cortico-cortical disconnections due to demyelination or a strict disruption of the motor network caused by neurodegenerative processes (60). Since NIBS has been widely used as rehabilitation treatments for neurological diseases and various mechanisms based on neuroplasticity, neural protection and/or regeneration induced by NIBS are being explored, adaptation of NIBS protocols according to different stages of brain damage and degeneration await further research (61).

## Strengths and limitations

To achieve the best possible results, we searched the literature extensively and evaluated outcome indicators comprehensively. We found that the estimates of treatment effects in our study were mostly in line with previous reviews on the same topic, but by combining direct and indirect evidence through NMA, we were able to increase the accuracy of the estimates. Furthermore, the main findings of our analysis were based on the strength of evidence to highlight the most robust findings.

It should be noted, however, that our review has limitations. All the included studies concluded that the available evidence had a very low level of quality due to bias, indirectness and inconsistency, thereby reducing their degree of certainty. In addition, the heterogeneity of clinical and radiological features of MS makes it difficult to determine which distinctive NIBS pattern would be most suitable for all patients. MS symptoms and disabilities are associated with asymmetric interhemispheric brain excitability (62), typically caused by neuroinflammation at its onset or degeneration as it progresses (63). Both cortical demyelination and subcortical myelination contribute to motor cortex excitability, resulting in variability in plasticity-inducing effects of NIBS between individuals (64, 65). The possibility of detecting dysfunctional cortical circuits, monitoring the disease course and developing novel neuromodulatory interventions represents a significant gap in the literature that needs to be addressed (66).

Therefore, current results need to be interpreted with caution. To improve the methodological rigor of the evidence, more RCTs with a higher level of quality, more participants, and a longer follow-up period are needed. Furthermore, adaptation of NIBS protocols according to different stages of brain damage in PwMS await further research.

## Conclusion

Together, our findings presented further evidence that NIBS might be promising and effective treatment for fatigue, spasticity, pain and QOL in pwMS. However, large, well-designed RCTs should be conducted to further validate the findings.

## Author contributions

XZ conceived the study. XZ, YH, ZW, WY, QX, and LY contributed to the study design. XZ drafted the manuscript. YH and LY edited the manuscript. All authors read and approved the final manuscript.

## Funding

This work was supported by the Shenzhen Longhua District Rehabilitation Medical Equipment Development and Transformation Joint Key Laboratory.

## Conflict of interest

The authors declare that the research was conducted in the absence of any commercial or financial relationships that could be construed as a potential conflict of interest.

## Publisher's note

All claims expressed in this article are solely those of the authors and do not necessarily represent those of their affiliated organizations, or those of the publisher, the editors and the reviewers. Any product that may be evaluated in this article, or claim that may be made by its manufacturer, is not guaranteed or endorsed by the publisher.

## Supplementary material

Supplementary Figure S1 Network diagrams of the immediate outcomes: **(A)** accuracy, **(B)** reaction time, **(C)** fatigue, **(D)** manual dexterity, **(E)** pain, **(F)** QOL, **(G)** spasticity.

Supplementary Figure S2 Network diagrams of the follow-up effects: **(A)** fatigue, **(B)** spasticity.

Supplementary Figure S3 Funnel plot on publication bias of immediate effect: **(A)** accuracy, **(B)** reaction time, **(C)** fatigue, **(D)** manual dexterity, **(E)** pain, **(F)** QOL, **(G)** spasticity.

Supplementary Figure S4 Funnel plot on publication bias of Longer durable effects: **(A)** fatigue, **(B)** spasticity.

Supplementary Table 1 Search items and search details.

Supplementary Table 2 Standardized pre-defined data extraction form.

Supplementary Table 3 League table of comparisons the immediate effect between all interventions for outcome parameters: **(A)** accuracy, **(B)** reaction time, **(C)** fatigue, **(D)** manual dexterity, **(E)** pain, **(F)** QOL, **(G)** spasticity. The certainty of the evidence (according to GRADE) was incorporated in this figure. ^*^Very low quality of evidence.

Supplementary Table 4 League table of comparisons the follow-up effect between all interventions for outcome parameters: **(A)** fatigue, **(B)** spasticity. The certainty of the evidence (according to GRADE) was incorporated in this figure. ^*^Very low quality of evidence.

Supplementary Table 5 Number of studies and patients involved in each neurological domain.
