# Supplementary figures and images for: Non-invasive brain stimulation therapy on neurological symptoms in patients with multiple sclerosis: A network meta analysis
Source: Front Neurol. 2022 Nov 15;13:1007702. doi: 10.3389/fneur.2022.1007702 (PMC9705977; doi:10.3389/fneur.2022.1007702)

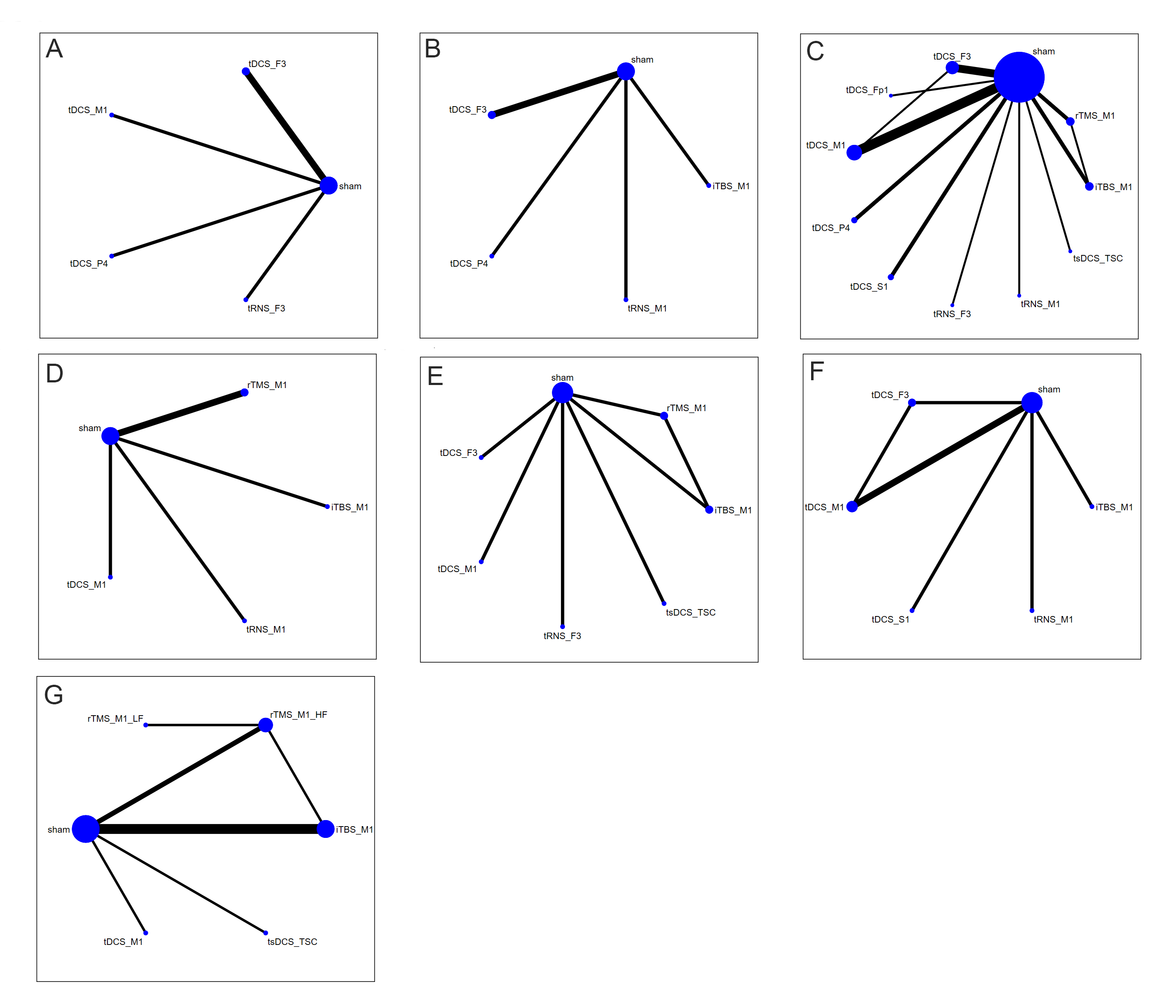

Supplement: Supplementary Figure S1 — Network diagrams of the immediate outcomes: (A) accuracy, (B) reaction time, (C) fatigue, (D) manual dexterity, (E) pain, (F) QOL, (G) spasticity. [file Image_1.TIF]

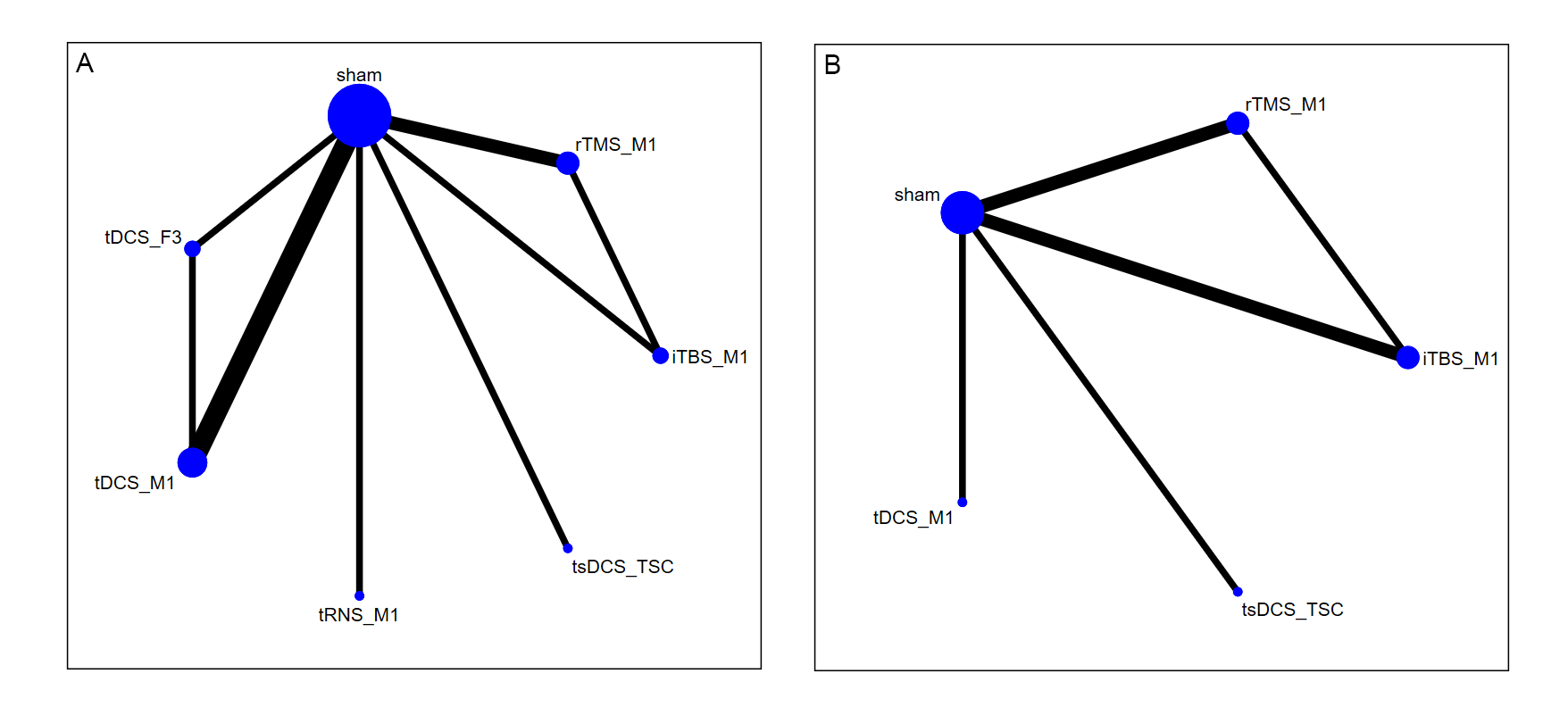

Supplement: Supplementary Figure S2 — Network diagrams of the follow-up effects: (A) fatigue, (B) spasticity. [file Image_2.TIF]

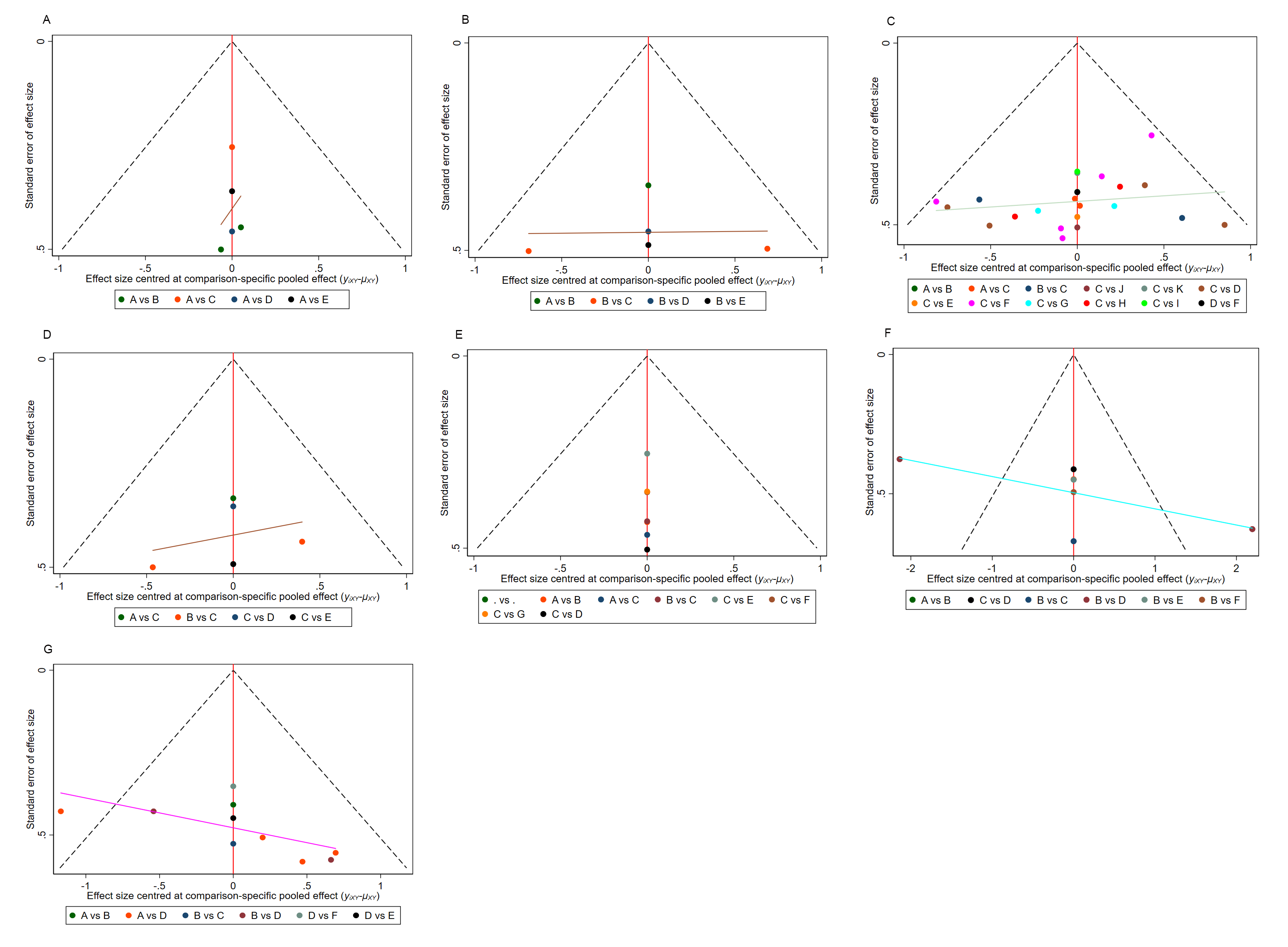

Supplement: Supplementary Figure S3 — Funnel plot on publication bias of immediate effect: (A) accuracy, (B) reaction time, (C) fatigue, (D) manual dexterity, (E) pain, (F) QOL, (G) spasticity. [file Image_3.TIF]

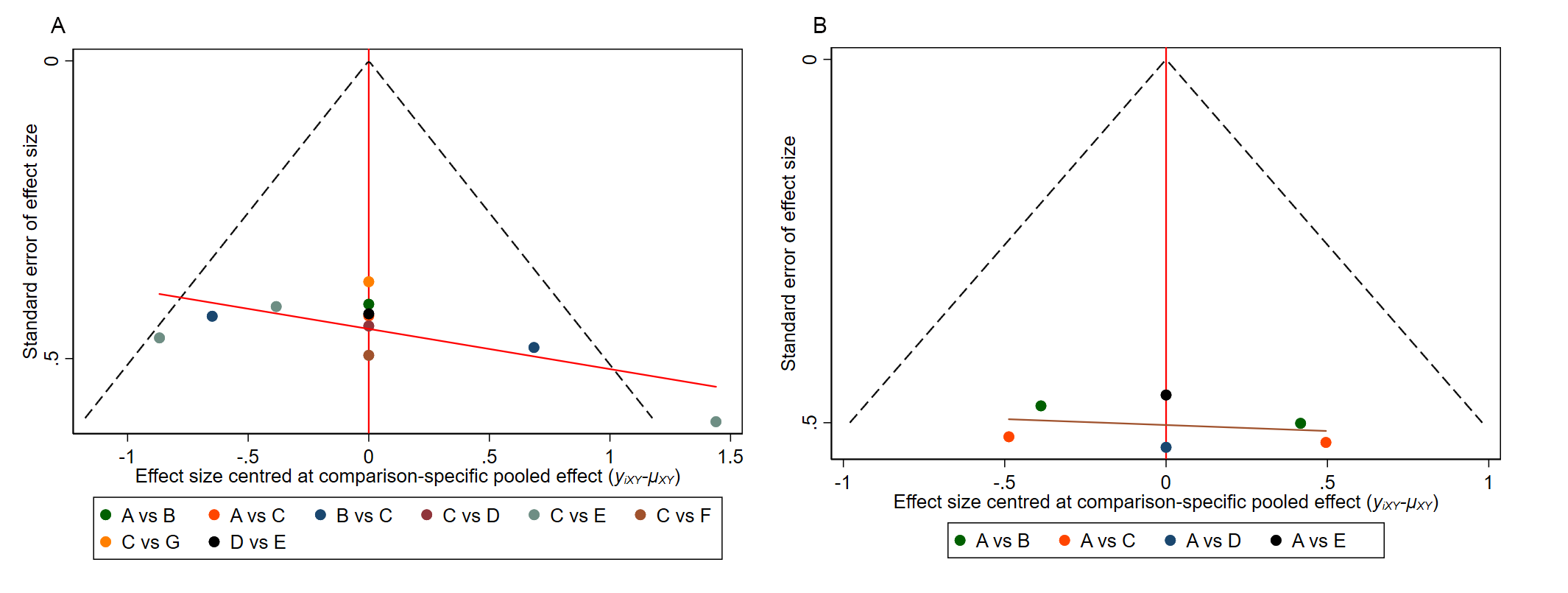

Supplement: Supplementary Figure S4 — Funnel plot on publication bias of Longer durable effects: (A) fatigue, (B) spasticity. [file Image_4.TIF]
