# Supplementary material for: Non-invasive brain stimulation therapy on neurological symptoms in patients with multiple sclerosis: A network meta analysis
Source: Front Neurol. 2022 Nov 15;13:1007702. doi: 10.3389/fneur.2022.1007702 (PMC9705977; doi:10.3389/fneur.2022.1007702)
Supplement: Supplementary Table 1 — Search items and search details. [file Table_1.docx]

**Supplementary Table 1.** Search Items and Search Details

**Pubmed**

| Search number | Search Details | Results |
| --- | --- | --- |
| 1 | "Multiple Sclerosis"[MeSH Terms] | 65,197 |
| 2 | "multiple sclerosis"[Title/Abstract] OR "sclerosis multiple"[Title/Abstract] OR "sclerosis disseminated"[Title/Abstract] OR "disseminated sclerosis"[Title/Abstract] OR (("ms"[Journal] OR "med sci paris"[Journal] OR "ms"[All Fields]) AND "multiple sclerosis"[Title/Abstract]) OR "multiple sclerosis acute fulminating"[Title/Abstract] | 84,582 |
| 3 | "Multiple Sclerosis"[MeSH Terms] OR ("Multiple Sclerosis"[Title/Abstract] OR "sclerosis multiple"[Title/Abstract] OR "sclerosis disseminated"[Title/Abstract] OR "disseminated sclerosis"[Title/Abstract] OR (("ms"[Journal] OR "med sci paris"[Journal] OR "ms"[All Fields]) AND "Multiple Sclerosis"[Title/Abstract]) OR "multiple sclerosis acute fulminating"[Title/Abstract]) | 92,133 |
| 4 | "Transcranial Direct Current Stimulation"[MeSH Terms] | 3,951 |
| 5 | "transcranial direct current stimulation"[Title/Abstract] OR "tDCS"[Title/Abstract] OR ("Cathodal"[All Fields] AND "stimulation transcranial direct current stimulation"[Title/Abstract]) OR "cathodal stimulation tdcs"[Title/Abstract] OR "stimulation tdcs cathodal"[Title/Abstract] OR "tdcs cathodal stimulation"[Title/Abstract] OR "transcranial random noise stimulation"[Title/Abstract] OR "transcranial alternating current stimulation"[Title/Abstract] OR "transcranial electrical stimulation"[Title/Abstract] OR "electrical stimulation transcranial"[Title/Abstract] OR "stimulation transcranial electrical"[Title/Abstract] OR "anodal stimulation transcranial direct current stimulation"[Title/Abstract] OR "anodal stimulation tdcs"[Title/Abstract] OR "stimulation tdcs anodal"[Title/Abstract] OR "tdcs anodal stimulation"[Title/Abstract] OR "repetitive transcranial electrical stimulation"[Title/Abstract] | 7,391 |
| 6 | "Transcranial Direct Current Stimulation"[MeSH Terms] OR ("Transcranial Direct Current Stimulation"[Title/Abstract] OR "tDCS"[Title/Abstract] OR ("Cathodal"[All Fields] AND "stimulation transcranial direct current stimulation"[Title/Abstract]) OR "cathodal stimulation tdcs"[Title/Abstract] OR "stimulation tdcs cathodal"[Title/Abstract] OR "tdcs cathodal stimulation"[Title/Abstract] OR "transcranial random noise stimulation"[Title/Abstract] OR "transcranial alternating current stimulation"[Title/Abstract] OR "transcranial electrical stimulation"[Title/Abstract] OR "electrical stimulation transcranial"[Title/Abstract] OR "stimulation transcranial electrical"[Title/Abstract] OR "anodal stimulation transcranial direct current stimulation"[Title/Abstract] OR "anodal stimulation tdcs"[Title/Abstract] OR "stimulation tdcs anodal"[Title/Abstract] OR "tdcs anodal stimulation"[Title/Abstract] OR "repetitive transcranial electrical stimulation"[Title/Abstract]) | 7,779 |
| 7 | "Transcranial Magnetic Stimulation"[MeSH Terms] | 13,405 |
| 8 | "transcranial magnetic stimulation"[Title/Abstract] OR "magnetic stimulation transcranial"[Title/Abstract] OR "stimulation transcranial magnetic"[Title/Abstract] OR "transcranial magnetic stimulations"[Title/Abstract] OR "transcranial magnetic stimulation single pulse"[Title/Abstract] OR "transcranial magnetic stimulation paired pulse"[Title/Abstract] OR "transcranial magnetic stimulation repetitive"[Title/Abstract] | 16,957 |
| 9 | "Transcranial Magnetic Stimulation"[MeSH Terms] OR "Transcranial Magnetic Stimulation"[Title/Abstract] OR "magnetic stimulation transcranial"[Title/Abstract] OR "stimulation transcranial magnetic"[Title/Abstract] OR "transcranial magnetic stimulations"[Title/Abstract] OR "transcranial magnetic stimulation single pulse"[Title/Abstract] OR "transcranial magnetic stimulation paired pulse"[Title/Abstract] OR "transcranial magnetic stimulation repetitive"[Title/Abstract] | 19,376 |
| 10 | "Electroconvulsive Therapy"[MeSH Terms] | 13,758 |
| 11 | "electroconvulsive therapy"[Title/Abstract] OR "therapy electroconvulsive"[Title/Abstract] OR "electroshock therapy"[Title/Abstract] OR "therapy electroshock"[Title/Abstract] OR "convulsive therapy electric"[Title/Abstract] OR "electric convulsive therapy"[Title/Abstract] OR (("therapeutics"[MeSH Terms] OR "therapeutics"[All Fields] OR "Therapies"[All Fields] OR "Therapy"[MeSH Subheading] OR "Therapy"[All Fields] OR "therapy s"[All Fields] OR "therapys"[All Fields]) AND "electric convulsive"[Title/Abstract]) OR "shock therapy electric"[Title/Abstract] OR "electric shock therapies"[Title/Abstract] OR (("Shock"[MeSH Terms] OR "Shock"[All Fields] OR "shocked"[All Fields] OR "shocking"[All Fields] OR "shocks"[All Fields]) AND "therapies electric"[Title/Abstract]) OR (("therapeutics"[MeSH Terms] OR "therapeutics"[All Fields] OR "Therapies"[All Fields] OR "Therapy"[MeSH Subheading] OR "Therapy"[All Fields] OR "therapy s"[All Fields] OR "therapys"[All Fields]) AND "electric shock"[Title/Abstract]) OR ("ECT"[All Fields] AND "Psychotherapy"[Title/Abstract]) | 11,019 |
| 12 | "electroconvulsive therapies"[Title/Abstract] OR "therapies electroconvulsive"[Title/Abstract] OR "electroshock therapies"[Title/Abstract] OR (("therapeutics"[MeSH Terms] OR "therapeutics"[All Fields] OR "Therapies"[All Fields] OR "Therapy"[MeSH Subheading] OR "Therapy"[All Fields] OR "therapy s"[All Fields] OR "therapys"[All Fields]) AND "Electroshock"[Title/Abstract]) OR (("convulsants"[Pharmacological Action] OR "convulsants"[MeSH Terms] OR "convulsants"[All Fields] OR "convulsant"[All Fields] OR "convulse"[All Fields] OR "convulsed"[All Fields] OR "convulsing"[All Fields] OR "Convulsive"[All Fields] OR "convulsives"[All Fields] OR "seizures"[MeSH Terms] OR "seizures"[All Fields] OR "convulsion"[All Fields] OR "convulsions"[All Fields]) AND "therapies electric"[Title/Abstract]) OR "electric convulsive therapies"[Title/Abstract] OR (("therapeutics"[MeSH Terms] OR "therapeutics"[All Fields] OR "Therapies"[All Fields] OR "Therapy"[MeSH Subheading] OR "Therapy"[All Fields] OR "therapy s"[All Fields] OR "therapys"[All Fields]) AND "electric convulsive"[Title/Abstract]) OR (("Shock"[MeSH Terms] OR "Shock"[All Fields] OR "shocked"[All Fields] OR "shocking"[All Fields] OR "shocks"[All Fields]) AND "therapies electric"[Title/Abstract]) OR "electric shock therapies"[Title/Abstract] OR "shock therapy electric"[Title/Abstract] OR (("therapeutics"[MeSH Terms] OR "therapeutics"[All Fields] OR "Therapies"[All Fields] OR "Therapy"[MeSH Subheading] OR "Therapy"[All Fields] OR "therapy s"[All Fields] OR "therapys"[All Fields]) AND "electric shock"[Title/Abstract]) OR ("ECT"[All Fields] AND "Psychotherapies"[Title/Abstract]) | 5,018 |
| 13 | "electroconvulsive therapy"[Title/Abstract] OR "therapy electroconvulsive"[Title/Abstract] OR "electroshock therapy"[Title/Abstract] OR "therapy electroshock"[Title/Abstract] OR "convulsive therapy electric"[Title/Abstract] OR "electric convulsive therapy"[Title/Abstract] OR "therapy electric convulsive"[Title/Abstract] OR "shock therapy electric"[Title/Abstract] OR "electric shock therapy"[Title/Abstract] OR "shock therapy electric"[Title/Abstract] OR "therapy electric shock"[Title/Abstract] OR ("ECT"[All Fields] AND "Psychotherapy"[Title/Abstract]) | 10,125 |
| 14 | "Electroconvulsive Therapy"[MeSH Terms] OR ("Electroconvulsive Therapy"[Title/Abstract] OR "therapy electroconvulsive"[Title/Abstract] OR "electroshock therapy"[Title/Abstract] OR "therapy electroshock"[Title/Abstract] OR "convulsive therapy electric"[Title/Abstract] OR "electric convulsive therapy"[Title/Abstract] OR (("therapeutics"[MeSH Terms] OR "therapeutics"[All Fields] OR "Therapies"[All Fields] OR "Therapy"[MeSH Subheading] OR "Therapy"[All Fields] OR "therapy s"[All Fields] OR "therapys"[All Fields]) AND "electric convulsive"[Title/Abstract]) OR "shock therapy electric"[Title/Abstract] OR "electric shock therapies"[Title/Abstract] OR (("Shock"[MeSH Terms] OR "Shock"[All Fields] OR "shocked"[All Fields] OR "shocking"[All Fields] OR "shocks"[All Fields]) AND "therapies electric"[Title/Abstract]) OR (("therapeutics"[MeSH Terms] OR "therapeutics"[All Fields] OR "Therapies"[All Fields] OR "Therapy"[MeSH Subheading] OR "Therapy"[All Fields] OR "therapy s"[All Fields] OR "therapys"[All Fields]) AND "electric shock"[Title/Abstract]) OR ("ECT"[All Fields] AND "Psychotherapy"[Title/Abstract])) | 16,922 |
| 15 | "transcranial focused ultrasound stimulation"[Title/Abstract] OR "cranial electrotherapy stimulation"[Title/Abstract] OR "reduced impedance non invasive cortical electrostimulation"[Title/Abstract] OR "non invasive stimulation"[Title/Abstract] OR "transcutaneous electrical nerve stimulation"[Title/Abstract] OR "transcranial pulse stimulation"[Title/Abstract] OR "non invasive brain stimulation"[Title/Abstract] | 4,066 |
| 16 | "Transcranial Direct Current Stimulation"[MeSH Terms] OR ("Transcranial Direct Current Stimulation"[Title/Abstract] OR "tDCS"[Title/Abstract] OR ("Cathodal"[All Fields] AND "stimulation transcranial direct current stimulation"[Title/Abstract]) OR "cathodal stimulation tdcs"[Title/Abstract] OR "stimulation tdcs cathodal"[Title/Abstract] OR "tdcs cathodal stimulation"[Title/Abstract] OR "transcranial random noise stimulation"[Title/Abstract] OR "transcranial alternating current stimulation"[Title/Abstract] OR "transcranial electrical stimulation"[Title/Abstract] OR "electrical stimulation transcranial"[Title/Abstract] OR "stimulation transcranial electrical"[Title/Abstract] OR "anodal stimulation transcranial direct current stimulation"[Title/Abstract] OR "anodal stimulation tdcs"[Title/Abstract] OR "stimulation tdcs anodal"[Title/Abstract] OR "tdcs anodal stimulation"[Title/Abstract] OR "repetitive transcranial electrical stimulation"[Title/Abstract]) OR ("Transcranial Magnetic Stimulation"[MeSH Terms] OR ("Transcranial Magnetic Stimulation"[Title/Abstract] OR "magnetic stimulation transcranial"[Title/Abstract] OR "stimulation transcranial magnetic"[Title/Abstract] OR "transcranial magnetic stimulations"[Title/Abstract] OR "transcranial magnetic stimulation single pulse"[Title/Abstract] OR "transcranial magnetic stimulation paired pulse"[Title/Abstract] OR "transcranial magnetic stimulation repetitive"[Title/Abstract])) OR ("Electroconvulsive Therapy"[MeSH Terms] OR ("Electroconvulsive Therapy"[Title/Abstract] OR "therapy electroconvulsive"[Title/Abstract] OR "electroshock therapy"[Title/Abstract] OR "therapy electroshock"[Title/Abstract] OR "convulsive therapy electric"[Title/Abstract] OR "electric convulsive therapy"[Title/Abstract] OR (("therapeutics"[MeSH Terms] OR "therapeutics"[All Fields] OR "Therapies"[All Fields] OR "Therapy"[MeSH Subheading] OR "Therapy"[All Fields] OR "therapy s"[All Fields] OR "therapys"[All Fields]) AND "electric convulsive"[Title/Abstract]) OR "shock therapy electric"[Title/Abstract] OR "electric shock therapies"[Title/Abstract] OR (("Shock"[MeSH Terms] OR "Shock"[All Fields] OR "shocked"[All Fields] OR "shocking"[All Fields] OR "shocks"[All Fields]) AND "therapies electric"[Title/Abstract]) OR (("therapeutics"[MeSH Terms] OR "therapeutics"[All Fields] OR "Therapies"[All Fields] OR "Therapy"[MeSH Subheading] OR "Therapy"[All Fields] OR "therapy s"[All Fields] OR "therapys"[All Fields]) AND "electric shock"[Title/Abstract]) OR ("ECT"[All Fields] AND "Psychotherapy"[Title/Abstract]))) OR ("transcranial focused ultrasound stimulation"[Title/Abstract] OR "cranial electrotherapy stimulation"[Title/Abstract] OR "reduced impedance non invasive cortical electrostimulation"[Title/Abstract] OR "non invasive stimulation"[Title/Abstract] OR "transcutaneous electrical nerve stimulation"[Title/Abstract] OR "transcranial pulse stimulation"[Title/Abstract] OR "non invasive brain stimulation"[Title/Abstract]) | 44,116 |
| 17 | ("Multiple Sclerosis"[MeSH Terms] OR ("Multiple Sclerosis"[Title/Abstract] OR "sclerosis multiple"[Title/Abstract] OR "sclerosis disseminated"[Title/Abstract] OR "disseminated sclerosis"[Title/Abstract] OR (("ms"[Journal] OR "med sci paris"[Journal] OR "ms"[All Fields]) AND "Multiple Sclerosis"[Title/Abstract]) OR "multiple sclerosis acute fulminating"[Title/Abstract])) AND ("Transcranial Direct Current Stimulation"[MeSH Terms] OR ("Transcranial Direct Current Stimulation"[Title/Abstract] OR "tDCS"[Title/Abstract] OR ("Cathodal"[All Fields] AND "stimulation transcranial direct current stimulation"[Title/Abstract]) OR "cathodal stimulation tdcs"[Title/Abstract] OR "stimulation tdcs cathodal"[Title/Abstract] OR "tdcs cathodal stimulation"[Title/Abstract] OR "transcranial random noise stimulation"[Title/Abstract] OR "transcranial alternating current stimulation"[Title/Abstract] OR "transcranial electrical stimulation"[Title/Abstract] OR "electrical stimulation transcranial"[Title/Abstract] OR "stimulation transcranial electrical"[Title/Abstract] OR "anodal stimulation transcranial direct current stimulation"[Title/Abstract] OR "anodal stimulation tdcs"[Title/Abstract] OR "stimulation tdcs anodal"[Title/Abstract] OR "tdcs anodal stimulation"[Title/Abstract] OR "repetitive transcranial electrical stimulation"[Title/Abstract]) OR ("Transcranial Magnetic Stimulation"[MeSH Terms] OR ("Transcranial Magnetic Stimulation"[Title/Abstract] OR "magnetic stimulation transcranial"[Title/Abstract] OR "stimulation transcranial magnetic"[Title/Abstract] OR "transcranial magnetic stimulations"[Title/Abstract] OR "transcranial magnetic stimulation single pulse"[Title/Abstract] OR "transcranial magnetic stimulation paired pulse"[Title/Abstract] OR "transcranial magnetic stimulation repetitive"[Title/Abstract])) OR ("Electroconvulsive Therapy"[MeSH Terms] OR ("Electroconvulsive Therapy"[Title/Abstract] OR "therapy electroconvulsive"[Title/Abstract] OR "electroshock therapy"[Title/Abstract] OR "therapy electroshock"[Title/Abstract] OR "convulsive therapy electric"[Title/Abstract] OR "electric convulsive therapy"[Title/Abstract] OR (("therapeutics"[MeSH Terms] OR "therapeutics"[All Fields] OR "Therapies"[All Fields] OR "Therapy"[MeSH Subheading] OR "Therapy"[All Fields] OR "therapy s"[All Fields] OR "therapys"[All Fields]) AND "electric convulsive"[Title/Abstract]) OR "shock therapy electric"[Title/Abstract] OR "electric shock therapies"[Title/Abstract] OR (("Shock"[MeSH Terms] OR "Shock"[All Fields] OR "shocked"[All Fields] OR "shocking"[All Fields] OR "shocks"[All Fields]) AND "therapies electric"[Title/Abstract]) OR (("therapeutics"[MeSH Terms] OR "therapeutics"[All Fields] OR "Therapies"[All Fields] OR "Therapy"[MeSH Subheading] OR "Therapy"[All Fields] OR "therapy s"[All Fields] OR "therapys"[All Fields]) AND "electric shock"[Title/Abstract]) OR ("ECT"[All Fields] AND "Psychotherapy"[Title/Abstract]))) OR ("transcranial focused ultrasound stimulation"[Title/Abstract] OR "cranial electrotherapy stimulation"[Title/Abstract] OR "reduced impedance non invasive cortical electrostimulation"[Title/Abstract] OR "non invasive stimulation"[Title/Abstract] OR "transcutaneous electrical nerve stimulation"[Title/Abstract] OR "transcranial pulse stimulation"[Title/Abstract] OR "non invasive brain stimulation"[Title/Abstract])) | 427 |
| 18 | (("Multiple Sclerosis"[MeSH Terms] OR ("Multiple Sclerosis"[Title/Abstract] OR "sclerosis multiple"[Title/Abstract] OR "sclerosis disseminated"[Title/Abstract] OR "disseminated sclerosis"[Title/Abstract] OR (("ms"[Journal] OR "med sci paris"[Journal] OR "ms"[All Fields]) AND "Multiple Sclerosis"[Title/Abstract]) OR "multiple sclerosis acute fulminating"[Title/Abstract])) AND ("Transcranial Direct Current Stimulation"[MeSH Terms] OR ("Transcranial Direct Current Stimulation"[Title/Abstract] OR "tDCS"[Title/Abstract] OR ("Cathodal"[All Fields] AND "stimulation transcranial direct current stimulation"[Title/Abstract]) OR "cathodal stimulation tdcs"[Title/Abstract] OR "stimulation tdcs cathodal"[Title/Abstract] OR "tdcs cathodal stimulation"[Title/Abstract] OR "transcranial random noise stimulation"[Title/Abstract] OR "transcranial alternating current stimulation"[Title/Abstract] OR "transcranial electrical stimulation"[Title/Abstract] OR "electrical stimulation transcranial"[Title/Abstract] OR "stimulation transcranial electrical"[Title/Abstract] OR "anodal stimulation transcranial direct current stimulation"[Title/Abstract] OR "anodal stimulation tdcs"[Title/Abstract] OR "stimulation tdcs anodal"[Title/Abstract] OR "tdcs anodal stimulation"[Title/Abstract] OR "repetitive transcranial electrical stimulation"[Title/Abstract]) OR ("Transcranial Magnetic Stimulation"[MeSH Terms] OR ("Transcranial Magnetic Stimulation"[Title/Abstract] OR "magnetic stimulation transcranial"[Title/Abstract] OR "stimulation transcranial magnetic"[Title/Abstract] OR "transcranial magnetic stimulations"[Title/Abstract] OR "transcranial magnetic stimulation single pulse"[Title/Abstract] OR "transcranial magnetic stimulation paired pulse"[Title/Abstract] OR "transcranial magnetic stimulation repetitive"[Title/Abstract])) OR ("Electroconvulsive Therapy"[MeSH Terms] OR ("Electroconvulsive Therapy"[Title/Abstract] OR "therapy electroconvulsive"[Title/Abstract] OR "electroshock therapy"[Title/Abstract] OR "therapy electroshock"[Title/Abstract] OR "convulsive therapy electric"[Title/Abstract] OR "electric convulsive therapy"[Title/Abstract] OR (("therapeutics"[MeSH Terms] OR "therapeutics"[All Fields] OR "Therapies"[All Fields] OR "Therapy"[MeSH Subheading] OR "Therapy"[All Fields] OR "therapy s"[All Fields] OR "therapys"[All Fields]) AND "electric convulsive"[Title/Abstract]) OR "shock therapy electric"[Title/Abstract] OR "electric shock therapies"[Title/Abstract] OR (("Shock"[MeSH Terms] OR "Shock"[All Fields] OR "shocked"[All Fields] OR "shocking"[All Fields] OR "shocks"[All Fields]) AND "therapies electric"[Title/Abstract]) OR (("therapeutics"[MeSH Terms] OR "therapeutics"[All Fields] OR "Therapies"[All Fields] OR "Therapy"[MeSH Subheading] OR "Therapy"[All Fields] OR "therapy s"[All Fields] OR "therapys"[All Fields]) AND "electric shock"[Title/Abstract]) OR ("ECT"[All Fields] AND "Psychotherapy"[Title/Abstract]))) OR ("transcranial focused ultrasound stimulation"[Title/Abstract] OR "cranial electrotherapy stimulation"[Title/Abstract] OR "reduced impedance non invasive cortical electrostimulation"[Title/Abstract] OR "non invasive stimulation"[Title/Abstract] OR "transcutaneous electrical nerve stimulation"[Title/Abstract] OR "transcranial pulse stimulation"[Title/Abstract] OR "non invasive brain stimulation"[Title/Abstract]))) AND (clinicaltrial[Filter]) | 63 |

**WOS search items**

| Search number | Search Details | Results |
| --- | --- | --- |
| #1 | Multiple Sclerosis (Topic) or Sclerosis, Multiple (Topic) or Sclerosis, Disseminated (Topic) or MS (Multiple Sclerosis) (Topic) or Multiple Sclerosis, Acute Fulminating (Topic) | [132849](https://0-www.webofscience.com.carlson.utoledo.edu/wos/woscc/summary/42b84cf5-b1b1-4220-943a-1432e6415668-240edf87/relevance/1) |
| #2 | Transcranial Direct Current Stimulation (Topic) or Transcranial Random Noise Stimulation (Topic) or Transcranial Alternating Current Stimulation (Topic) or Transcranial Electrical Stimulation (Topic) or Transcranial Magnetic Stimulation (Topic) or Electroconvulsive Therapy (Topic) or transcranial focused ultrasound stimulation (Topic) or cranial electrotherapy stimulation (Topic) or reduced impedance non-invasive cortical electrostimulation (Topic) or non-invasive stimulation (Topic) or transcutaneous electrical nerve stimulation (Topic) or transcranial pulse stimulation (Topic) or non-invasive brain stimulation (Topic) | [43907](https://0-www.webofscience.com.carlson.utoledo.edu/wos/woscc/summary/2bcf0d3e-f2e7-4ed4-bb9b-7cd4badd62db-240f094a/relevance/1) |
| #3 | #1 AND #2 | [721](https://0-www.webofscience.com.carlson.utoledo.edu/wos/woscc/summary/332b31b4-7575-4751-826c-92e3f9da65ea-240f1f1f/relevance/1) |

**Embase queries**

| No. | Query | Results |
| --- | --- | --- |
| #12 | #3 AND #11 | 861 |
| #11 | #4 OR #5 OR #6 OR #7 OR #8 OR #9 OR #10 | 63614 |
| #10 | 'transcranial focused ultrasound stimulation':ab,ti OR 'cranial electrotherapy stimulation':ab,ti OR 'reduced impedance non-invasive cortical electrostimulation':ab,ti OR 'non-invasive stimulation':ab,ti OR 'transcutaneous electrical nerve stimulation':ab,ti OR 'transcranial pulse stimulation':ab,ti OR 'non-invasive brain stimulation':ab,ti | 5226 |
| #9 | 'electroconvulsive therapy':ab,ti OR 'therapies, electroconvulsive':ab,ti OR 'electroshock therapy':ab,ti OR 'therapies, electroshock':ab,ti OR 'convulsive therapy, electric':ab,ti OR 'electric convulsive therapies':ab,ti OR 'therapies, electric convulsive':ab,ti OR 'shock therapy, electric':ab,ti OR 'electric shock therapies':ab,ti OR 'shock therapies, electric':ab,ti OR 'therapies, electric shock':ab,ti OR (ect:ab,ti AND psychotherapy:ab,ti) | 10980 |
| #8 | 'electroconvulsive therapy'/exp | 22332 |
| #7 | 'transcranial magnetic stimulation':ab,ti OR 'magnetic stimulation, transcranial':ab,ti OR 'magnetic stimulations, transcranial':ab,ti OR 'stimulation, transcranial magnetic':ab,ti OR 'stimulations, transcranial magnetic':ab,ti OR 'transcranial magnetic stimulations':ab,ti OR 'transcranial magnetic stimulation, single pulse':ab,ti OR 'transcranial magnetic stimulation, paired pulse':ab,ti OR 'transcranial magnetic stimulation, repetitive':ab,ti | 22596 |
| #6 | 'transcranial magnetic stimulation'/exp | 27695 |
| #5 | 'transcranial direct current stimulation':ab,ti OR tdcs:ab,ti OR 'cathodal stimulation transcranial direct current stimulation':ab,ti OR 'cathodal stimulation tdcs':ab,ti OR 'stimulation tdcs, cathodal':ab,ti OR 'tdcs, cathodal stimulation':ab,ti OR 'transcranial random noise stimulation':ab,ti OR 'transcranial alternating current stimulation':ab,ti OR 'transcranial electrical stimulation':ab,ti OR 'electrical stimulation, transcranial':ab,ti OR 'stimulation, transcranial electrical':ab,ti OR 'transcranial electrical stimulations':ab,ti OR 'anodal stimulation transcranial direct current stimulation':ab,ti OR 'anodal stimulation tdcs':ab,ti OR 'stimulation tdcs, anodal':ab,ti OR 'tdcs, anodal stimulation':ab,ti OR 'repetitive transcranial electrical stimulation':ab,ti | 10283 |
| #4 | 'transcranial direct current stimulation'/exp | 8941 |
| #3 | #1 OR #2 | 160144 |
| #2 | 'multiple sclerosis':ab,ti OR 'sclerosis, multiple':ab,ti OR 'sclerosis, disseminated':ab,ti OR 'disseminated sclerosis':ab,ti OR (ms:ab,ti AND 'multiple sclerosis':ab,ti) OR 'multiple sclerosis, acute fulminating':ab,ti | 130745 |
| #1 | 'multiple sclerosis'/exp | 147357 |

**Ovid MEDLINE**

| [#](https://ovidsp.dc2.ovid.com/ovid-a/ovidweb.cgi?&S=JFALFPHINPEBBPKPJPNJCGDGNBOEAA00&Sort+Sets=descending) | **Searches** | **Results** |
| --- | --- | --- |
| 1 | exp Multiple Sclerosis/ | 208277 |
|  |  |  |
| 2 | exp Transcranial Direct Current Stimulation/ | 12887 |
|  |  |  |
| 3 | exp Transcranial Magnetic Stimulation/ | 41100 |
|  |  |  |
| 4 | exp Electroconvulsive Therapy/ | 33462 |
|  |  |  |
| 5 | Multiple Sclerosis.ab,at. | 201587 |
|  |  |  |
| 6 | Transcranial Direct Current Stimulation.ab,at. | 12874 |
|  |  |  |
| 7 | Transcranial Magnetic Stimulation.ab,at. | 40378 |
|  |  |  |
| 8 | Electroconvulsive Therapy.ab,at. | 17483 |
|  |  |  |
| 9 | Transcranial Random Noise Stimulation.ab,at. | 356 |
|  |  |  |
| 10 | Transcranial Alternating Current Stimulation.ab,at. | 1271 |
|  |  |  |
| 11 | Transcranial Electrical Stimulation.ab,at. | 1489 |
|  |  |  |
| 12 | transcranial focused ultrasound stimulation.ab,at. | 38 |
|  |  |  |
| 13 | cranial electrotherapy stimulation.ab,at. | 172 |
|  |  |  |
| 14 | reduced impedance non-invasive cortical electrostimulation.ab,at. | 8 |
|  |  |  |
| 15 | non-invasive stimulation.ab,at. | 462 |
|  |  |  |
| 16 | transcutaneous electrical nerve stimulation.ab,at. | 5156 |
|  |  |  |
| 17 | transcranial pulse stimulation.ab,at. | 13 |
|  |  |  |
| 18 | non-invasive brain stimulation.ab,at. | 3114 |
|  |  |  |
| 19 | 1 or 5 | 266785 |
|  |  |  |
| 20 | 2 or 3 or 4 or 6 or 7 or 8 or 9 or 10 or 11 or 12 or 13 or 14 or 15 or 16 or 17 or 18 | 109258 |
|  |  |  |
| 21 | 19 and 20 | 1324 |
|  |  |  |
| 22 | 21 use medall | 403 |
|  |  |  |

**Cochrane**

| #1 | MeSH descriptor: [Multiple Sclerosis] explode all trees | MeSH |  | 3831 |
| --- | --- | --- | --- | --- |
| #2 | (Multiple Sclerosis):ti,ab,kw OR (Sclerosis, Multiple):ti,ab,kw OR (Sclerosis, Disseminated):ti,ab,kw OR (Disseminated Sclerosis):ti,ab,kw OR (MS (Multiple Sclerosis)):ti,ab,kw | S | Limits | 11331 |
| #3 | (Multiple Sclerosis, Acute Fulminating):ti,ab,kw | S | Limits | 0 |
| #4 | MeSH descriptor: [Transcranial Direct Current Stimulation] explode all trees | MeSH |  | 914 |
| #5 | (Transcranial Direct Current Stimulation):ti,ab,kw OR (tDCS):ti,ab,kw OR (Cathodal Stimulation Transcranial Direct Current Stimulation):ti,ab,kw OR (Cathodal Stimulation tDCS):ti,ab,kw OR (Stimulation tDCS, Cathodal):ti,ab,kw | S | Limits | 4986 |
| #6 | (tDCS, Cathodal Stimulation):ti,ab,kw OR (Transcranial Random Noise Stimulation):ti,ab,kw OR (Transcranial Alternating Current Stimulation):ti,ab,kw OR (Transcranial Electrical Stimulation):ti,ab,kw OR (Electrical Stimulation, transcranial) ):ti,ab,kw | S | Limits | 1915 |
| #7 | (Stimulation, Transcranial Electrical):ti,ab,kw OR (Transcranial Electrical Stimulations):ti,ab,kw OR (Anodal Stimulation Transcranial Direct Current Stimulation):ti,ab,kw OR (Anodal Stimulation tDCS):ti,ab,kw OR (Stimulation tDCS, Anodal):ti,ab,kw | S | Limits | 2760 |
| #8 | (tDCS, Anodal Stimulation):ti,ab,kw OR (Repetitive Transcranial Electrical Stimulation):ti,ab,kw | S | Limits | 1971 |
| #9 | MeSH descriptor: [Transcranial Magnetic Stimulation] explode all trees | MeSH |  | 1569 |
| #10 | (Transcranial Magnetic Stimulation):ti,ab,kw OR (Magnetic Stimulation, Transcranial):ti,ab,kw OR (Stimulation, Transcranial Magnetic):ti,ab,kw OR (Transcranial Magnetic Stimulations):ti,ab,kw OR (Transcranial Magnetic Stimulation, Single Pulse):ti,ab,kw | S | Limits | 5945 |
| #11 | (Transcranial Magnetic Stimulation, Paired Pulse):ti,ab,kw OR (Transcranial Magnetic Stimulation, Repetitive):ti,ab,kw | S | Limits | 3453 |
| #12 | MeSH descriptor: [Electroconvulsive Therapy] explode all trees | MeSH |  | 607 |
| #13 | (Electroconvulsive Therapy):ti,ab,kw OR (Therapies, Electroconvulsive):ti,ab,kw OR (Electroshock Therapy):ti,ab,kw OR (Therapies, Electroshock):ti,ab,kw OR (Convulsive Therapy, Electric):ti,ab,kw | S | Limits | 1719 |
| #14 | (Electric Convulsive Therapies):ti,ab,kw OR (Therapies, Electric Convulsive):ti,ab,kw OR (Shock Therapy, Electric):ti,ab,kw OR (Electric Shock Therapies):ti,ab,kw OR (Shock Therapies, Electric):ti,ab,kw | S | Limits | 366 |
| #15 | (Therapies, Electric Shock):ti,ab,kw OR (ECT (Psychotherapy)):ti,ab,kw OR (transcranial focused ultrasound stimulation):ti,ab,kw OR (cranial electrotherapy stimulation):ti,ab,kw OR (reduced impedance non invasive cortical electrostimulation):ti,ab,kw | S | Limits | 149 |
| #16 | (non-invasive stimulation):ti,ab,kw OR (transcutaneous electrical nerve stimulation):ti,ab,kw OR (transcranial pulse stimulation):ti,ab,kw OR (non-invasive brain stimulation):ti,ab,kw | S | Limits | 5298 |
| #17 | #1 OR #2 OR #3 |  | Limits | 11331 |
| #18 | #4 OR #5 OR #6 OR #7 OR #8 OR #9 OR #10 OR #11 OR #12 OR #13 OR #14 OR #15 OR #16 |  | Limits | 15753 |
| #19 | #17 AND #18 |  | Limits | 221 |
