# Supplementary material for: Non-invasive brain stimulation therapy on neurological symptoms in patients with multiple sclerosis: A network meta analysis
Source: Front Neurol. 2022 Nov 15;13:1007702. doi: 10.3389/fneur.2022.1007702 (PMC9705977; doi:10.3389/fneur.2022.1007702)
Supplement: Supplementary Table 2 — Standardized pre-defined data extraction form. [file Table_2.docx]

**Supplementary Table 2. S**tandardised pre-defined data extraction form

| Author | Year of publication | Country or region | Sample size | Male/Female | Age (mean+SD) | Intervention | Control | Outcome indicators | Experiment group | | |
| --- | --- | --- | --- | --- | --- | --- | --- | --- | --- | --- | --- |
|  |  |  |  |  |  |  |  |  | Electrode placement | Intensity | Area |
|  |  |  |  |  |  |  |  |  |  |  |  |
|  |  |  |  |  |  |  |  |  |  |  |  |
|  |  |  |  |  |  |  |  |  |  |  |  |
|  |  |  |  |  |  |  |  |  |  |  |  |
|  |  |  |  |  |  |  |  |  |  |  |  |
|  |  |  |  |  |  |  |  |  |  |  |  |
|  |  |  |  |  |  |  |  |  |  |  |  |
|  |  |  |  |  |  |  |  |  |  |  |  |
|  |  |  |  |  |  |  |  |  |  |  |  |
