# Supplementary material for: Non-invasive brain stimulation therapy on neurological symptoms in patients with multiple sclerosis: A network meta analysis
Source: Front Neurol. 2022 Nov 15;13:1007702. doi: 10.3389/fneur.2022.1007702 (PMC9705977; doi:10.3389/fneur.2022.1007702)
Supplement: Supplementary Table 3 — League table of comparisons the immediate effect between all interventions for outcome parameters: (A) accuracy, (B) reaction time, (C) fatigue, (D) manual dexterity, (E) pain, (F) QOL, (G) spasticity. The certainty of the evidence (according to GRADE) was incorporated in this figure. *Very low quality of evidence. [file Table_3.docx]

**Supplementary Table 3.** League table of comparisons the immediate effect between all interventions for outcome parameters: 3A. accuracy, 3B. reaction time, 3C. fatigue, 3D. manual dexterity, 3E. pain, 3F. QOL, 3G. spasticity. The certainty of the evidence (according to GRADE) was incorporated in this figure. *Very low quality of evidence.

**3A. accuracy**

| tRNS_F3 | tDCS_P4 | tDCS_M1 | sham | tDCS_F3 |
| --- | --- | --- | --- | --- |
| tRNS_F3 | 0.01^*^ (-1.13,1.15) | -0.40^*^ (-1.26,0.47) | -0.53^*^ (-1.23,0.18) | -0.58^*^ (-1.54,0.38) |
| -0.01^*^ (-1.15,1.13) | tDCS_P4 | -0.41^*^ (-1.43,0.62) | -0.54^*^ (-1.44,0.35) | -0.59^*^ (-1.70,0.52) |
| 0.40^*^ (-0.47,1.26) | 0.41^*^ (-0.62,1.43) | tDCS_M1 | -0.13^*^ (-0.63,0.37) | -0.18^*^ (-1.01,0.64) |
| 0.53^*^ (-0.18,1.23) | 0.54^*^ (-0.35,1.44) | 0.13^*^ (-0.37,0.63) | sham | -0.05^*^ (-0.70,0.60) |
| 0.58^*^ (-0.38,1.54) | 0.59^*^ (-0.52,1.70) | 0.18^*^ (-0.64,1.01) | 0.05^*^ (-0.60,0.70) | tDCS_F3 |

**3B.** **reaction time**

| tRNS_M1 | iTBS_M1 | sham | tDCS_PPC | tDCS_F3 |
| --- | --- | --- | --- | --- |
| tRNS_M1 | 0.07^*^ (-2.53,2.67) | 0.17^*^ (-1.73,2.07) | 0.61^*^ (-2.05,3.27) | 0.67^*^ (-1.66,3.00) |
| -0.07^*^ (-2.67,2.53) | iTBS_M1 | 0.10^*^ (-1.67,1.87) | 0.54^*^ (-2.03,3.12) | 0.60^*^ (-1.63,2.82) |
| -0.17^*^ (-2.07,1.73) | -0.10^*^ (-1.87,1.67) | sham | 0.44^*^ (-1.42,2.31) | 0.50^*^ (-0.85,1.85) |
| -0.61^*^ (-3.27,2.05) | -0.54^*^ (-3.12,2.03) | -0.44^*^ (-2.31,1.42) | tDCS_PPC | 0.05^*^ (-2.25,2.36) |
| -0.67^*^ (-3.00,1.66) | -0.60^*^ (-2.82,1.63) | -0.50^*^ (-1.85,0.85) | -0.05^*^ (-2.36,2.25) | tDCS_F3 |

**3C. fatigue**

| rTMS_M1 | tDCS_F3 | tRNS_M1 | tDCS_S1 | tsDCS_TSC | tDCS_P4 | tDCS_M1 | iTBS_M1 | tRNS_F3 | sham | tDCS_Fp1 |
| --- | --- | --- | --- | --- | --- | --- | --- | --- | --- | --- |
| rTMS_M1 | 0.19^*^ (-0.69,1.07) | 0.13^*^ (-1.22,1.48) | 0.27^*^ (-0.75,1.29) | 0.39^*^ (-0.76,1.55) | 0.45^*^ (-0.59,1.48) | 0.52^*^ (-0.31,1.36) | 0.60^*^ (-0.23,1.42) | 0.93^*^ (-0.22,2.08) | 0.85^*^ (0.14,1.57) | 1.57^*^ (0.25,2.88) |
| -0.19^*^ (-1.07,0.69) | tDCS_F3 | -0.06^*^ (-1.31,1.20) | 0.09^*^ (-0.80,0.97) | 0.21^*^ (-0.83,1.24) | 0.26^*^ (-0.64,1.17) | 0.34^*^ (-0.27,0.95) | 0.41^*^ (-0.46,1.28) | 0.74^*^ (-0.29,1.77) | 0.67^*^ (0.16,1.18) | 1.38^*^ (0.17,2.59) |
| -0.13^*^ (-1.48,1.22) | 0.06^*^ (-1.20,1.31) | tRNS_M1 | 0.14^*^ (-1.21,1.49) | 0.26^*^ (-1.20,1.72) | 0.32^*^ (-1.05,1.68) | 0.39^*^ (-0.83,1.62) | 0.46^*^ (-0.88,1.81) | 0.80^*^ (-0.66,2.25) | 0.72^*^ (-0.42,1.87) | 1.43^*^ (-0.15,3.02) |
| -0.27^*^ (-1.29,0.75) | -0.09^*^ (-0.97,0.80) | -0.14^*^ (-1.49,1.21) | tDCS_S1 | 0.12^*^ (-1.03,1.28) | 0.18^*^ (-0.86,1.22) | 0.25^*^ (-0.59,1.09) | 0.32^*^ (-0.68,1.33) | 0.66^*^ (-0.50,1.81) | 0.58^*^ (-0.14,1.30) | 1.29^*^ (-0.02,2.61) |
| -0.39^*^ (-1.55,0.76) | -0.21^*^ (-1.24,0.83) | -0.26^*^ (-1.72,1.20) | -0.12^*^ (-1.28,1.03) | tsDCS_TSC | 0.05^*^ (-1.12,1.23) | 0.13^*^ (-0.87,1.13) | 0.20^*^ (-0.94,1.35) | 0.53^*^ (-0.74,1.81) | 0.46^*^ (-0.44,1.36) | 1.17^*^ (-0.25,2.59) |
| -0.45^*^ (-1.48,0.59) | -0.26^*^ (-1.17,0.64) | -0.32^*^ (-1.68,1.05) | -0.18^*^ (-1.22,0.86) | -0.05^*^ (-1.23,1.12) | tDCS_P4 | 0.08^*^ (-0.79,0.94) | 0.15^*^ (-0.88,1.17) | 0.48^*^ (-0.69,1.65) | 0.41^*^ (-0.34,1.15) | 1.12^*^ (-0.21,2.44) |
| -0.52^*^ (-1.36,0.31) | -0.34^*^ (-0.95,0.27) | -0.39^*^ (-1.62,0.83) | -0.25^*^ (-1.09,0.59) | -0.13^*^ (-1.13,0.87) | -0.08^*^ (-0.94,0.79) | tDCS_M1 | 0.07^*^ (-0.75,0.90) | 0.40^*^ (-0.59,1.40) | 0.33^*^ (-0.10,0.76) | 1.04^*^ (-0.14,2.22) |
| -0.60^*^ (-1.42,0.23) | -0.41^*^ (-1.28,0.46) | -0.46^*^ (-1.81,0.88) | -0.32^*^ (-1.33,0.68) | -0.20^*^ (-1.35,0.94) | -0.15^*^ (-1.17,0.88) | -0.07^*^ (-0.90,0.75) | iTBS_M1 | 0.33^*^ (-0.81,1.47) | 0.26^*^ (-0.44,0.96) | 0.97^*^ (-0.33,2.27) |
| -0.93^*^ (-2.08,0.22) | -0.74^*^ (-1.77,0.29) | -0.80^*^ (-2.25,0.66) | -0.66^*^ (-1.81,0.50) | -0.53^*^ (-1.81,0.74) | -0.48^*^ (-1.65,0.69) | -0.40^*^ (-1.40,0.59) | -0.33^*^ (-1.47,0.81) | tRNS_F3 | -0.07^*^ (-0.97,0.82) | 0.64^*^ (-0.78,2.05) |
| -0.85^*^ (-1.57,-0.14) | -0.67^*^ (-1.18,-0.16) | -0.72^*^ (-1.87,0.42) | -0.58^*^ (-1.30,0.14) | -0.46^*^ (-1.36,0.44) | -0.41^*^ (-1.15,0.34) | -0.33^*^ (-0.76,0.10) | -0.26^*^ (-0.96,0.44) | 0.07^*^ (-0.82,0.97) | sham | 0.71^*^ (-0.39,1.81) |
| -1.57^*^ (-2.88,-0.25) | -1.38^*^ (-2.59,-0.17) | -1.43^*^ (-3.02,0.15) | -1.29^*^ (-2.61,0.02) | -1.17^*^ (-2.59,0.25) | -1.12^*^ (-2.44,0.21) | -1.04^*^ (-2.22,0.14) | -0.97^*^ (-2.27,0.33) | -0.64^*^ (-2.05,0.78) | -0.71^*^ (-1.81,0.39) | tDCS_Fp1 |

**3D. manual dexterity**

| rTMS_M1 | iTBS_M1 | sham | tDCS_C3 | tRNS_M1 |
| --- | --- | --- | --- | --- |
| rTMS_M1 | 0.36^*^ (-0.96,1.67) | 0.50^*^ (-0.35,1.35) | 0.58^*^ (-0.75,1.91) | 0.90^*^ (-0.59,2.39) |
| -0.36^*^ (-1.67,0.96) | iTBS_M1 | 0.14^*^ (-0.86,1.15) | 0.22^*^ (-1.21,1.66) | 0.54^*^ (-1.05,2.13) |
| -0.50^*^ (-1.35,0.35) | -0.14^*^ (-1.15,0.86) | sham | 0.08^*^ (-0.95,1.11) | 0.40^*^ (-0.83,1.63) |
| -0.58^*^ (-1.91,0.75) | -0.22^*^ (-1.66,1.21) | -0.08^*^ (-1.11,0.95) | tDCS_C3 | 0.32^*^ (-1.28,1.92) |
| -0.90^*^ (-2.39,0.59) | -0.54^*^ (-2.13,1.05) | -0.40^*^ (-1.63,0.83) | -0.32^*^ (-1.92,1.28) | tRNS_M1 |

**3E. pain**

| iTBS_M1 | tDCS_M1 | tDCS_F3 | tsDCS_TSC | sham | rTMS_M1 | tRNS_F3 |
| --- | --- | --- | --- | --- | --- | --- |
| iTBS_M1 | 1.08^*^ (0.23,1.93) | 1.10^*^ (-0.25,2.44) | 1.20^*^ (0.05,2.35) | 1.26^*^ (0.11,2.40) | 1.39^*^ (0.48,2.30) | 1.47^*^ (0.42,2.51) |
| -1.08^*^ (-1.93,-0.23) | tDCS_M1 | 0.02^*^ (-1.28,1.32) | 0.12^*^ (-0.97,1.21) | 0.18^*^ (-0.91,1.27) | 0.31^*^ (-0.53,1.15) | 0.38^*^ (-0.60,1.36) |
| -1.10^*^ (-2.44,0.25) | -0.02^*^ (-1.32,1.28) | tDCS_F3 | 0.10^*^ (-1.10,1.31) | 0.16^*^ (-1.04,1.37) | 0.29^*^ (-0.70,1.28) | 0.37^*^ (-0.74,1.47) |
| -1.20^*^ (-2.35,-0.05) | -0.12^*^ (-1.21,0.97) | -0.10^*^ (-1.31,1.10) | tsDCS_TSC | 0.06^*^ (-0.92,1.04) | 0.19^*^ (-0.51,0.88) | 0.26^*^ (-0.59,1.12) |
| -1.26^*^ (-2.40,-0.11) | -0.18^*^ (-1.27,0.91) | -0.16^*^ (-1.37,1.04) | -0.06^*^ (-1.04,0.92) | sham | 0.13^*^ (-0.56,0.82) | 0.21^*^ (-0.65,1.06) |
| -1.39^*^ (-2.30,-0.48) | -0.31^*^ (-1.15,0.53) | -0.29^*^ (-1.28,0.70) | -0.19^*^ (-0.88,0.51) | -0.13^*^ (-0.82,0.56) | rTMS_M1 | 0.07^*^ (-0.42,0.57) |
| -1.47^*^ (-2.51,-0.42) | -0.38^*^ (-1.36,0.60) | -0.37^*^ (-1.47,0.74) | -0.26^*^ (-1.12,0.59) | -0.21^*^ (-1.06,0.65) | -0.07^*^ (-0.57,0.42) | tRNS_F3 |

**3F. QOL**

| tDCS_F3 | tRNS_M1 | tDCS_S1 | tDCS_M1 | sham | iTBS_M1 |
| --- | --- | --- | --- | --- | --- |
| tDCS_F3 | -0.96^*^ (-2.32,0.40) | -1.16^*^ (-2.46,0.14) | -0.89^*^ (-1.69,-0.10) | -1.41^*^ (-2.36,-0.45) | -1.25^*^ (-2.55,0.04) |
| 0.96^*^ (-0.40,2.32) | tRNS_M1 | -0.20^*^ (-1.51,1.11) | 0.07^*^ (-1.09,1.23) | -0.44^*^ (-1.41,0.52) | -0.29^*^ (-1.60,1.02) |
| 1.16^*^ (-0.14,2.46) | 0.20^*^ (-1.11,1.51) | tDCS_S1 | 0.27^*^ (-0.82,1.35) | -0.25^*^ (-1.13,0.63) | -0.09^*^ (-1.34,1.15) |
| 0.89^*^ (0.10,1.69) | -0.07^*^ (-1.23,1.09) | -0.27^*^ (-1.35,0.82) | tDCS_M1 | -0.51^*^ (-1.15,0.12) | -0.36^*^ (-1.44,0.72) |
| 1.41^*^ (0.45,2.36) | 0.44^*^ (-0.52,1.41) | 0.25^*^ (-0.63,1.13) | 0.51^*^ (-0.12,1.15) | sham | 0.15^*^ (-0.72,1.03) |
| 1.25^*^ (-0.04,2.55) | 0.29^*^ (-1.02,1.60) | 0.09^*^ (-1.15,1.34) | 0.36^*^ (-0.72,1.44) | -0.15^*^ (-1.03,0.72) | iTBS_M1 |

**3G. spasticity**

| iTBS_M1 | rTMS_M1_HF | tDCS_M1 | tsDCS_TSC | sham | rTMS_M1_LF |
| --- | --- | --- | --- | --- | --- |
| iTBS_M1 | 0.37^*^ (-0.78,1.51) | 1.00^*^ (-0.71,2.70) | 1.24^*^ (-0.38,2.85) | 1.20^*^ (0.41,1.99) | 2.01^*^ (0.03,3.98) |
| -0.37^*^ (-1.51,0.78) | rTMS_M1_HF | 0.63^*^ (-1.21,2.47) | 0.87^*^ (-0.89,2.63) | 0.83^*^ (-0.21,1.88) | 1.64^*^ (0.03,3.25) |
| -1.00^*^ (-2.70,0.71) | -0.63^*^ (-2.47,1.21) | tDCS_M1 | 0.24^*^ (-1.83,2.31) | 0.20^*^ (-1.31,1.72) | 1.01^*^ (-1.43,3.46) |
| -1.24^*^ (-2.85,0.38) | -0.87^*^ (-2.63,0.89) | -0.24^*^ (-2.31,1.83) | tsDCS_TSC | -0.04^*^ (-1.45,1.38) | 0.77^*^ (-1.61,3.15) |
| -1.20^*^ (-1.99,-0.41) | -0.83^*^ (-1.88,0.21) | -0.20^*^ (-1.72,1.31) | 0.04^*^ (-1.38,1.45) | sham | 0.81^*^ (-1.11,2.73) |
| -2.01^*^ (-3.98,-0.03) | -1.64^*^ (-3.25,-0.03) | -1.01^*^ (-3.46,1.43) | -0.77^*^ (-3.15,1.61) | -0.81^*^ (-2.73,1.11) | rTMS_M1_LF |
