# Supplementary material for: Non-invasive brain stimulation therapy on neurological symptoms in patients with multiple sclerosis: A network meta analysis
Source: Front Neurol. 2022 Nov 15;13:1007702. doi: 10.3389/fneur.2022.1007702 (PMC9705977; doi:10.3389/fneur.2022.1007702)
Supplement: Supplementary Table 4 — League table of comparisons the follow-up effect between all interventions for outcome parameters: (A) fatigue, (B) spasticity. The certainty of the evidence (according to GRADE) was incorporated in this figure. *Very low quality of evidence. [file Table_4.docx]

**Supplementary Table 4.** League table of comparisons the follow-up effect between all interventions for outcome parameters: 4A. fatigue, 4B. spasticity. The certainty of the evidence (according to GRADE) was incorporated in this figure. *Very low quality of evidence.

**4A**. **fatigue**

| tDCS_F3 | rTMS_M1 | tsDCS_TSC | tRNS_M1 | iTBS_M1 | tDCS_M1 | sham |
| --- | --- | --- | --- | --- | --- | --- |
| tDCS_F3 | 0.34^*^ (-2.09,2.78) | 0.31^*^ (-2.49,3.12) | 0.73^*^ (-2.15,3.61) | 0.81^*^ (-1.93,3.54) | 1.10^*^ (-0.81,3.02) | 1.19^*^ (-0.74,3.11) |
| -0.34^*^ (-2.78,2.09) | rTMS_M1 | -0.03^*^ (-2.56,2.50) | 0.39^*^ (-2.22,3.00) | 0.46^*^ (-1.48,2.41) | 0.76^*^ (-1.18,2.70) | 0.84^*^ (-0.65,2.33) |
| -0.31^*^ (-3.12,2.49) | 0.03^*^ (-2.50,2.56) | tsDCS_TSC | 0.42^*^ (-2.54,3.38) | 0.50^*^ (-2.33,3.32) | 0.79^*^ (-1.60,3.18) | 0.87^*^ (-1.17,2.92) |
| -0.73^*^ (-3.61,2.15) | -0.39^*^ (-3.00,2.22) | -0.42^*^ (-3.38,2.54) | tRNS_M1 | 0.08^*^ (-2.82,2.97) | 0.37^*^ (-2.10,2.85) | 0.46^*^ (-1.69,2.60) |
| -0.81^*^ (-3.54,1.93) | -0.46^*^ (-2.41,1.48) | -0.50^*^ (-3.32,2.33) | -0.08^*^ (-2.97,2.82) | iTBS_M1 | 0.30^*^ (-2.01,2.61) | 0.38^*^ (-1.57,2.33) |
| -1.10^*^ (-3.02,0.81) | -0.76^*^ (-2.70,1.18) | -0.79^*^ (-3.18,1.60) | -0.37^*^ (-2.85,2.10) | -0.30^*^ (-2.61,2.01) | tDCS_M1 | 0.08^*^ (-1.16,1.32) |
| -1.19^*^ (-3.11,0.74) | -0.84^*^ (-2.33,0.65) | -0.87^*^ (-2.92,1.17) | -0.46^*^ (-2.60,1.69) | -0.38^*^ (-2.33,1.57) | -0.08^*^ (-1.32,1.16) | sham |

**4B**. **spasticity**

| tsDCS_TSC | iTBS_M1 | tDCS_M1 | rTMS_M1 | sham |
| --- | --- | --- | --- | --- |
| tsDCS_TSC | 9.54^*^ (6.59,12.50) | 10.28^*^ (7.23,13.32) | 10.34^*^ (7.39,13.29) | 10.75^*^ (7.86,13.63) |
| -9.54^*^ (-12.50,-6.59) | iTBS_M1 | 0.73^*^ (-0.43,1.90) | 0.80^*^ (0.09,1.50) | 1.21^*^ (0.57,1.85) |
| -10.28^*^ (-13.32,-7.23) | -0.73^*^ (-1.90,0.43) | tDCS_M1 | 0.06^*^ (-1.09,1.22) | 0.47^*^ (-0.50,1.44) |
| -10.34^*^ (-13.29,-7.39) | -0.80^*^ (-1.50,-0.09) | -0.06^*^ (-1.22,1.09) | rTMS_M1 | 0.41^*^ (-0.21,1.03) |
| -10.75^*^ (-13.63,-7.86) | -1.21^*^ (-1.85,-0.57) | -0.47^*^ (-1.44,0.50) | -0.41^*^ (-1.03,0.21) | sham |
