# Supplementary material for: Non-invasive brain stimulation therapy on neurological symptoms in patients with multiple sclerosis: A network meta analysis
Source: Front Neurol. 2022 Nov 15;13:1007702. doi: 10.3389/fneur.2022.1007702 (PMC9705977; doi:10.3389/fneur.2022.1007702)
Supplement: Supplementary Table 5 — Number of studies and patients involved in each neurological domain. [file Table_5.docx]

**Supplementary Table 5.** Number of studies and patients involved in each neurological domain

|  | **Number of studies** | **Number of patients** | **Types and protocols of NIBS** | **References** |
| --- | --- | --- | --- | --- |
| **Immediate effect** | | | | |
| **Accuracy** | 4 | 73 | tDCS_F3, tDCS_M1, tDCS_P4, tRNS_F3 | Ayache 2016, Chalah 2017, Meesen 2014, Palm 2016 |
| **Reaction time** | 4 | 79 | iTBS_M1, tDCS_F3, tDCS_P4, tRNS_M1 | Azin 2016, Ayache 2016, Chalah 2017, Salemi 2019 |
| **Fatigue** | 17 | 396 | iTBS_M1, rTMS_M1, tDCS_F3, tDCS_Fp1, tDCS_M1, tDCS_P4, tDCS_S1, tRNS_F3, tRNS_M1, tsDCS_TSC | Ayache 2016, Berra 2019, Cancelli 2018, Chalah 2017, Charvet 2018, Elzamarany 2016, Ferrucci 2014, Gaede 2018, Korzhova 2019, Meesen 2014, Mori 2022, Mortezanejad 2020, Palm 2016, Pilloni 2020, Salemi 2019, Tecchio 2015, Hanken 2016 |
| **Manual dexterity** | 5 | 101 | iTBS_M1, rTMS_M1, tDCS_M1, tRNS_M1 | Azin 2016, Elzamarany 2016, Koch 2008, Salemi 2019, Seelmann-Eggebert 2021 |
| **Pain** | 5 | 130 | TSC, iTBS_M1, rTMS_M1, tDCS_F3, tDCS_M1, tRNS_F3 | Ayache 2016, Berra 2019, Korzhova 2019, Meesen 2014, Palm 2016 |
| **QOL** | 5 | 123 | iTBS_M1, tDCS_F3, tDCS_M1, tDCS_S1, tRNS_M1 | Young 2020, Mori 2011, Mori 2013, Mortezanejad 2020, Salemi 2019 |
| **Spasticity** | 9 | 198 | iTBS_M1, rTMS_M1_HF, rTMS_M1_LF, tDCS_M1, tsDCS_TSC | Abdelkader 2013, Berra 2019, Boutière 2017, Iodice 2015, Korzhova 2019, Mori 2010, Mori 2011, Şan 2019, Boutiere 2017 |
| **Longer durable effects** | | | | |
| **Fatigue** | 7 | 174 | iTBS_M1, rTMS_M1, tDCS_F3, tDCS_M1, tRNS_M1, tsDCS_TSC | Berra 2019, Elzamarany 2016, Ferrucci 2014, Korzhova 2019, Mortezanejad 2020, Pilloni 2020, Salemi 2019 |
| **Spasticity** | 5 | 120 | iTBS_M1, rTMS_M1, tDCS_M1, tDCS_TSC | Berra 2019, Boutière 2017, Korzhova 2019, Mori 2010, Şan 2019 |
